# Effect of Optimized Immunosuppression (Including Rituximab) on Anti-Donor Alloresponses in Patients With Chronically Rejecting Renal Allografts

**DOI:** 10.3389/fimmu.2020.00079

**Published:** 2020-02-05

**Authors:** Kin Yee Shiu, Dominic Stringer, Laura McLaughlin, Olivia Shaw, Paul Brookes, Hannah Burton, Hannah Wilkinson, Harriet Douthwaite, Tjir-Li Tsui, Adam Mclean, Rachel Hilton, Sian Griffin, Colin Geddes, Simon Ball, Richard Baker, Candice Roufosse, Catherine Horsfield, Anthony Dorling

**Affiliations:** ^1^Department of Inflammation Biology, MRC Centre for Transplantation, Guy's Hospital, King's College London, London, United Kingdom; ^2^Biostatistics and Health Informatics, The Institute of Psychiatry, Psychology and Neuroscience, King's College London, London, United Kingdom; ^3^Viapath Analytics LLP, London, United Kingdom; ^4^Histocompatibility and Immunogenetics, Imperial College Healthcare NHS Trust, London, United Kingdom; ^5^Imperial College Renal and Transplant Centre, Imperial College Healthcare NHS Trust, London, United Kingdom; ^6^Department of Nephrology and Transplantation, Guy's and St. Thomas' NHS Foundation Trust, London, United Kingdom; ^7^Department of Nephrology, University Hospital of Wales, Cardiff, United Kingdom; ^8^Renal Unit, Western Infirmary, NHS Greater Glasgow and Clyde Trust, Glasgow, United Kingdom; ^9^Department of Nephrology, University Hospital Birmingham, Birmingham, United Kingdom; ^10^Renal Unit, St. James's University Hospital, Leeds, United Kingdom; ^11^Department of Histopathology, Guy's and St. Thomas' NHS Foundation Trust, London, United Kingdom

**Keywords:** kidney transplantation, B lymphocytes, chronic rejection in renal transplant, rituximab, donor specific antibody (DSA)

## Abstract

RituxiCAN-C4 combined an open-labeled randomized controlled trial (RCT) in 7 UK centers to assess whether rituximab could stabilize kidney function in patients with chronic rejection, with an exploratory analysis of how B cell-depletion influenced T cell anti-donor responses relative to outcome. Between January 2007 and March 2015, 59 recruits were enrolled after screening, 23 of whom consented to the embedded RCT. Recruitment was halted when in a pre-specified per protocol interim analysis, the RCT was discovered to be significantly underpowered. This report therefore focuses on the exploratory analysis, in which we confirmed that when B cells promoted CD4+ anti-donor IFNγ production assessed by ELISPOT, this associated with inferior clinical outcome; these patterns were inhibited by optimized immunosuppression but not rituximab. B cell suppression of IFNγ production, which associated with number of transitional B cells and correlated with slower declines in kidney function was abolished by rituximab, which depleted transitional B cells for prolonged periods. We conclude that in this patient population, optimized immunosuppression but not rituximab promotes anti-donor alloresponses associated with favorable outcomes.

**Clinical Trial Registration:** Registered with EudraCT (2006-002330-38) and www.ClinicalTrials.gov, identifier: NCT00476164.

## Introduction

Late kidney allograft failure rates remain high (1, 2), such that ~3% of incident kidney transplant recipients return to dialysis each year (3). Immune-mediated injury is the single biggest cause (4), usually presenting as progressive dysfunction with histological features on biopsy of chronic antibody (Ab)-mediated rejection (CAMR) (5). Despite significant advances in our ability to recognize CAMR, there are still no widely established treatments.

The progressive decline in glomerular filtration rate (GFR) that precedes graft failure is highly variable (6–9), with many patients maintaining stable graft function for prolonged periods. The precise immunological factors that influence this rate of decline in GFR are unknown; differences in the IgG subclass of DSA (10) or the ability to fix complement (11) offer potential explanations. However, other factors associated with the presence of DSA might influence the progression of pathology, rate of functional deterioration and timing of eventual graft failure. There is significant debate within the field about the contribution of cell-mediated immune processes in CAMR (12). We've previously defined that B lymphocytes play a role in CAMR as antigen presenting cells (APC) for interferon-gamma (IFNγ) production by indirect pathway anti-donor T cells, revealed in Enzyme-Linked Immunosorbent Spot (ELISPOT) assays (13). Moreover, we also defined a significant association between ELISPOT patterns of anti-donor reactivity and changes in estimated (e)GFR (14). Importantly we showed that optimizing immunosuppression (IS), to influence anti-donor responses and suppress antigen presentation by B cells could stabilize graft function. These data suggested that B cell targeted therapy might have significant benefit in CAMR.

Rituximab is a monoclonal Ab that binds the CD20 antigen, expressed exclusively by B cells (but not plasma cells), resulting in depletion via a range of mechanisms (15). Licensed as a treatment for B cell lymphoma, it has been used successfully in autoimmune conditions, and at induction for kidney transplantation, particularly across ABO barriers (16). Early case reports of rituximab as a treatment for CAMR suggested a benefit in stabilizing eGFR (17–19), though with potentially serious infectious complications (20).

Post rituximab, circulating B cell numbers can take months to recover (21, 22), with some evidence of differential recovery of different B cell subpopulations (23–26). This includes some studies that show preferential recovery of transitional B cells, a B cell subpopulation that has been associated with immunological tolerance induction in autoimmunity and transplantation (27, 28). Therefore, using rituximab to disrupt antigen presentation seemed a logical approach to treat CAMR. In RituxiCAN-C4, we tested the hypothesis that B cell depletion would stabilize graft function and reduce proteinuria in patients who had failed to respond to a formal trial of optimized oral IS. We also used the trial as an opportunity to study the impact of optimized IS and rituximab on *in vitro* anti-donor IFNγ production, in association with its differential impact on B cell subpopulations.

## Materials and Methods

### Study Design and Participants

In this trial, only rituximab, used within the embedded investigator-led open-label randomized controlled trial (RCT), was treated as an investigational medicinal product. At the beginning of recruitment, eligible patients were >6 months post-transplantation, with eGFR >20 mL/min/1.73 m^2^ (by 4 variable Modification of Diet in Renal Disease equation), deteriorating kidney function [as defined by Dudley et al. (29) and confirmed by Cockcroft-Gault eGFR] and a for-cause biopsy within 3 months of recruitment, showing chronic allograft nephropathy by BANFF'97 criteria OR transplant glomerulopathy (TG), associated with diffuse linear C4d staining on ≥50% of peritubular (PTC) OR glomerular capillary endothelium, assessed by immunohistochemistry (IHC). Inclusion criteria were changed to improve recruitment, so that biopsy could be within 6 months of recruitment, performed for either a deteriorating eGFR or proteinuria (urinary protein creatinine ratio (PCR) ≥50 mg/mmol), and had to show either linear C4d on ≥25% of endothelium or PTCitis/glomerulitis with a combined PTC/g score of ≥2. None of these modifications were thought to alter the integrity of the trial. Biopsies were processed and interpreted locally. Each was re-interpreted according to latest BANFF criteria at study end. Exclusion criteria were (1) biopsy showing recurrent or *de novo* disease or calcineurin inhibitor (CNI) toxicity accompanied by supratherapeutic CNI trough levels, (2) <18 years old, (3) blood group incompatible or combined kidney/pancreas transplant or desensitization to remove HLA Ab prior to transplantation, (4) history of acute rejection, myocardial infarction, or administration of lymphocyte depleting Ab within 3 months of enrolment, (5) history of symptomatic ischaemic heart disease, or documented allergy to murine proteins and (6) history of a non-skin limited malignancy within 5 years. Post-consent screening was performed to exclude anyone with a positive HepBSAg, HepBcAb, HepCAb, HIV or HCG test (in females suspected to be pregnant) and those with ureteric obstruction on ultrasound scan.

Study conduct and patient safety was monitored by an independent data monitoring committee (DMC). Clinical coordination by the chief investigator (CI) was supported by the UK NIHR Clinical Research Networks. The study was approved by the MHRA and by the West London Committee of the National Research Ethics Service (06/Q0406/119) and was carried out in accordance with the declaration of Helsinki (1996) and Good Clinical Practice as defined in UK clinical trial regulations. All subjects gave written informed consent. The trial is registered with EudraCT (2006-002330-38) and with ClinicalTrials.gov (NCT00476164).

### Procedures (Figure 1)

Patients with eligible biopsies were approached for written informed consent. After eligibility testing, IS was optimized to twice daily mycophenolic acid (MPA) formulation (dose determined locally) and tacrolimus with target trough levels of 4–8 μg/L during phase 1 (0–2 months), followed by a 3-month observational period. Patients also took statins (target cholesterol ≤4.5 mmol/L) and ACE-I/ARB combination therapy (target BP ≤140/ ≤80). Optimized therapy was individually tailored and inability to tolerate one or more aspects was not classed as “failure.” Patients already deemed to be on optimal therapy went straight into the 3-month observation period.

**Figure 1:**
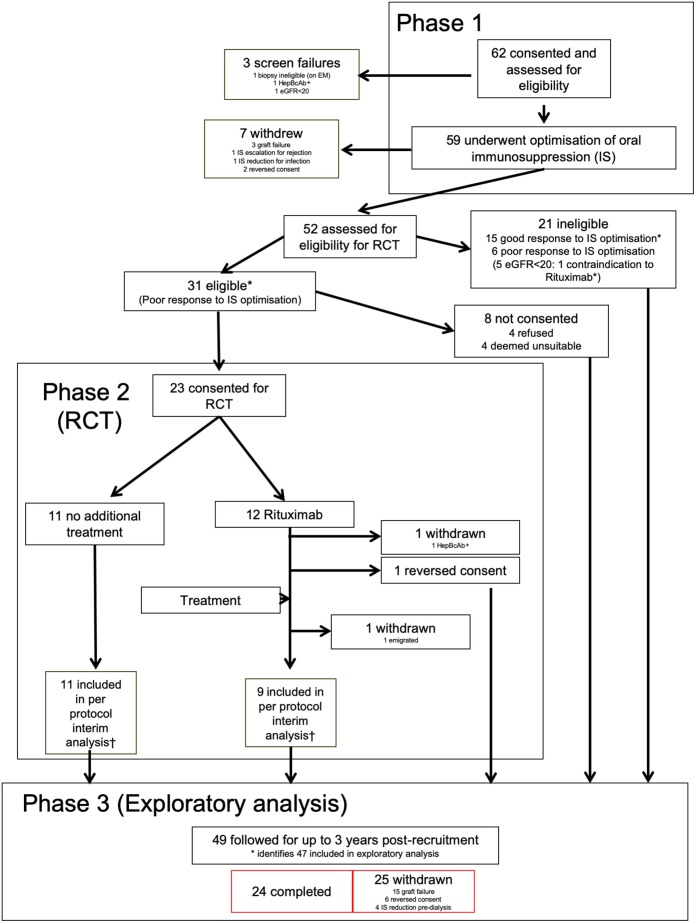
Consort diagram for RituxiCAN-C4 trial. *Indicates 47 patients included in the exploratory analysis. ^†^According to pre-specified second interim per protocol analysis.

At the end of phase 1, patients with an eGFR>20 mL/min/1.73 m^2^ and either a PCR ≥50 or continued deterioration of graft function were asked to consent to the RCT. Patients not meeting criteria and those who declined consent went to phase 3, where protocol-defined interventions ceased. Study observations that contributed to the exploratory study continued to 3 years post-recruitment. Any significant change in IS or graft failure in any phase were indications for withdrawal, including other treatments for chronic rejection such as plasmaphereis, IVIg or bortezomib.

### Design of the RCT

Detailed descriptions of the randomization process, blinding and interventions, primary and secondary objectives and end-points (EPs), effect size, sample size calculation for the RCT and statistical methodology are contained within the Supplemental Methods. Planned interim per protocol analyses (with stopping rules based primarily on adverse event frequency and secondarily on finding significant differences in response rates), were performed after recruitment of 36 (10 at primary EP) and 61 patients (20 at primary EP). Following the second interim analysis, the DMC halted further recruitment, as the trial was significantly underpowered.

### Statistical Analysis

Detailed explanation of the statistical analysis used in the RCT is contained in the Supplementary Material. For the exploratory analysis, we used Fisher exact probability, Mann-Whitney (30) or Kruskal-Wallis tests as appropriate. Data are presented as median ± IQR. A value of *P* < 0.05 was considered significant. The *P*-values are 2 sided, and because of the nature of the study, there are no adjustments for multiple comparisons.

### Exploratory Analysis Methodology

#### Calculation of ΔeGFR

Measured serum creatinines at all available time points were used to calculate eGFRs by 4 parameter MDRD equation, with appropriate correction for ethnicity, and these were used to generate equations describing the relationship between pre- and post-enrolment values, after normalizing the enrolment eGFR to zero. The ΔeGFRs generated by these equations avoided misinterpretation due to significant clinic to clinic variation in observed creatinines, If a patient suffered graft failure, or was withdrawn to prepare for dialysis, the ΔeGFR calculated for the day of graft failure or withdrawal was used for all subsequent time points. If a patient withdrew for other reasons, no data beyond the withdrawal date was used.

#### Anti-HLA Antibody Determination

Peripheral blood was obtained by standard phlebotomy in plain vacutainers (BD), and allowed to clot. Samples were centrifuged, and serum stored at −80°C until used. Analysis of anti-graft antibody was limited to donor specific antibodies (DSA) directed against HLA. Although tests for non-HLA DSA had been planned at the outset, these were not performed due to a shortage of funds. All HLA Ab testing was performed at a single laboratory (Guy's Hospital), which is a participant in the UK National Quality Assessment Service for Histocompatibility and Immunogenetics and uses their quality controls to validate the thresholds used for positive and negative antibody testing. Screening for HLA Ab was performed by flow cytometry using xMAP (Luminex) platform, utilizing LABScreen Mixed Bead. Positive samples were tested on single antigen HLA Class I and Class II kits (One Lambda, California, USA), used to further define specificity as described previously (14). No MFI cut off was applied for identification of DSA, though DSA with MFI <2000 are identified separately from those with MFI≥2000. Cumulative MFI was calculated for all DSA present, when more than one DSA was detected as previously described (31).

### Non-routine Laboratory Analysis

#### Preparation of Responder Peripheral Blood Mononuclear Cells (PBMC)

Peripheral blood samples were collected by standard phlebotomy (60 ml total volume), and processed within 8 h of venesection. PBMC were isolated by standard density gradient centrifugation using Lymphoprep (Axis-Shield, Stockport, UK). After washing, aliquots were frozen in 10% DMSO with 90% human AB serum (Life Technologies, Paisley, UK), and stored in liquid nitrogen until use. Magnetic bead separation was performed using CD8, CD19, and/or CD25 Dynabeads (Life Technologies); bound cells were discarded, and the negative fraction used in the ELISPOT assay. The composition of the resulting populations is illustrated in Supplementary Figure 1. Importantly, CD25 depletion resulted in complete loss of all CD25++ cells, but a significant number of CD25+ cells remained. All viable samples were analyzed by ELISPOT.

#### Interferon (IFN)-Gamma(γ) ELISPOT Assay

T cell responsiveness to alloantigens was assessed by IFNγ ELISPOT analysis as previously described (13, 14). The latter included, by necessity, flow cytometric analyses of T and B cell subsets to aid interpretation (see below). IFNγ ELISPOT plates (Mabtech AB, Nacka, Sweden) pre-coated with primary IFNγ Ab were blocked for 2 h with “complete medium” [AIM-V medium/10% human AB serum from Life Technologies)] before addition of 4 × 10^5^ responder PBMC per well in 100 μL of complete medium for 24 h at 37°C and 5% CO2, with either donor (or surrogate) proteins (at 100 and 500 ng/ml), a viral antigen cocktail to control for antigen processing and presentation; anti-CD3/anti-CD28–coated beads to control for cell viability and with media alone to control for background, as previously described (13). Each condition was performed in triplicate. Standardized operating procedures were followed. All counts were normalized to background and are reported as frequency of spot-forming cells/million CD4+ cells, where CD4+ cell percentages were determined by flow cytometry.

#### Preparation of Donor Antigens

PBMC were rapidly freeze-thawed three times using alternate liquid nitrogen/37°C water bath (32). The suspension was checked for lack of integrity of cells, ultracentrifuged at 100,000 g for 60 min at 4°C, then resuspended in solubilizing solution (6M urea, 2% CHAPS with protease inhibitor (Boehringer Mannheim, Bracknell, UK). Cells from the kidney donor were used where available, to provide the full array of HLA and non-HLA antigens.

Where donor material was not available, cytoplasmic membrane protein preparations were produced from surrogate donor cells obtained from HLA-typed healthy volunteers, splenocytes collected at the time of deceased donor donation at the Hammersmith and Guy's Hospitals in London, or from a collection of cytoplasmic membrane protein preparations obtained from tissue-typed donors as previously described (33). Appropriate surrogate donors were chosen according to the following hierarchy of rules; (i) shared as many as possible HLA-A, -B, -C, -DR, and -DQ mismatches with the actual donor and matched as many as possible recipient HLA; (ii) contained antigens that reflected the DSA profile of the recipient; (iii) contained no mismatches that reflected the non-DSA profile of the recipient; (iv) avoided mismatches from a previous failed transplant.

#### Flow Cytometry

PBMC were thawed, washed and then stained with titrated amounts of fluorochrome-conjugated monoclonal Ab in PBS with 10% human AB serum for 30 min at 4°C. Two panels were used. For T cells: CD4-FITC, CD25-PE, CD19-APC-Cy7, CD8-Qdot 605, CD14-Pacific blue, CD27-PerCP-Cy5.5, CD39-PE-Cy7. For B cells CD19-APC-Cy7, CD27-PerCPCy5.5, CD24-FITC, CD38-Qdot 605, CD14-Pacific blue. Ab were obtained from Ebioscience (San Diego, CA), BD Bioscience (Oxford, UK) and Life Technologies. Following staining, the cells were washed twice with PBS and then incubated with Fixable LIVE/DEAD Aqua-fluorescent reactive dye (Life Technologies) for 30 min at 4°C. Cells were washed, fixed for 15 min in 1% paraformaldehye, then washed with PBS-5% FCS and stored at 4°C before acquisition and analysis within 24 h on an LSRII/Fortessa flow cytometer at the BRC Flow Cytometry Laboratory, King's College London with Flowjo software (Treestar Inc). The gating strategy was identical to that previously reported (13). In brief, B cells were defined as CD19+ single cells within the living CD14-negative lymphocyte gate. CD27 was used on CD19+ cells to identify memory from non-memory cells. CD24 and CD38 were used on the CD27- population to distinguish naïve and transitional B cells. T1 and T2 were distinguished on the extent of CD38 and CD24 expression as defined by Cherukuri et al. (28). Tregs were identified by gating on CD25+ CD4+single cells within the CD14-negative lymphocyte gate and identifying high expression of CD39.

## Results

### Patients and Demographics

Between January 2007 and March 2015, 62 patients were recruited from seven UK centers (Supplementary Table 1). Of these, 3 were deemed ineligible after screening (Figure 1). The demographics of the remaining 59, including summary of biopsies are in Supplementary Table 2. Features of the 47 patients included in the exploratory analysis are summarized in Table 1. Individual enrolment biopsies are described in Supplementary Table 3. Trial follow-up completed in March 2017.

**Table 1:** Baseline characteristics of the patients included in the exploratory analysis.

	**Poor response to IS (*N* = 32)^a^**	**Good response to IS (*N* = 15)**	**P^b^**
**Age** [Years–Median (IQR)]	44 (22.7)	46 (13)	ø
**Male** *n* (%)	23 (72)	9 (60)	ø
**Ethnicity** ***n*** **(%)**			ø
Asian: Black: White	4(12.5): 3(9.4): 25(78.1)	2(13.3): 2(13.3): 11(74.3)	
**Cause of renal failure** ***n***			
DM	–	–	ø
APKD	2	–	ø
GN	10	7	ø
SLE	1	1	ø
HT	2	1	ø
Congenital^c^	8	1	ø
TIN^d^	5	1	ø
Cystinosis	2	–	ø
HUS	–	1	ø
CNI toxicity	–	1^e^	ø
Unknown/not recorded	2	2	ø
**Transplant history**			
Deceased: LRD: LURD	22: 8: 2	6: 6: 3	ø
Previous transplants: 0: 1	26: 6	14: 1	ø
Time from Tx [years-median (IQR)] HLA MM [Mean (*SD*)]	12.8 (14.4)	16.6 (12.7)	ø
Overall	2.9 (1.4)	3.3 (1)	ø
A: B:	1.1(0.6): 1.1(0.7)	1(0.8): 1.3(0.7)	ø
DR	0.7 (0.5)	1.2 (0.5)	*
**HLA Ab status**			
CRF [Mean (*SD*)]	48.1 (13.9)^g^	42.7 (37.7)	ø
DSA+ n (%)	17 (53)	11 (74.3)	ø
-Class I: Class II: Both	10(31): 3(9.7): 4(12.9)	3(20): 5(33.3): 3(20)	ø
-NA	15 (47)	4 (26.7)	ø
DSA MFI^f^ [Mean (*SD*)]	4437 (6627)	6758 (8998)	ø
**Enrolment biopsy scores**—**median**			
(IQR)			
C4d glom^h^	3 (1)	3 (1)	ø
Banff C4d (PTC)	2 (2)	2 (2)	ø
Bannf g	2 (1)	1(2)	ø
Banff ptc	1 (2)	1 (1)	ø
Banff cg	2 (2)	1 (1)	ø
Banff cv	1 (1)	1.5 (1)	ø
Banff ct	1 (1)	1 (1)	ø
Banff ci	1 (1)	1 (1)	ø
TA/IF (%)	25 (14)	20 (20)	ø
**Baseline immunosuppression** n (%)			
Tac: CsA	19 (59): 6 (19)	8 (53): 7 (47)	ø
MPA; Azathioprine	22 (69): 5 (16)	9 (60): 5 (33)	ø
**Baseline renal function** [Mean (*SD*)]			
Creatinine^i^	184.8 (51.7)	168.7 (44.8)	ø
eGFR^j^	37.7 (11.6)	38.6 (11.3)	ø
1/creat slope	−0.15 (0.23)	−0.07 (0.07)	ø
Formally deteriorating	23 (72)	10 (67)	ø
PCR^k^ [Mean (SD)]	213 (211)	74 (74)	†
PCR >50	27 (84)	8 (53)	*
**Post-optimization medication**			
Tac n (%)	31 (97)	15 (100)	ø
Tac level [Mean (SD)]	5.4 (2.7)	7.0 (2.2)	*
MPA n (%)	30 (94)	15 (100)	ø
MPA dose [mg (SD)]	953 (493)	1,000 (422)	ø
On ACE-I n (%)	20 (62.5)	6 (40)	ø
On ARB n (%)	22 (68.8)	12 (80)	ø

a *All who were eligible for RCT + the patient (G008) who developed a contraindication to Rituximab during phase 1*.

b *P value, comparing good response to optimized IS (N=15) to all poor response to optimized IS, eGFR>20 (N = 32)*.

c *Including Alports*.

d *Including chronic pyelonephritis*.

e *Heart transplant recipient*.

f *No HLA Ab data available on 1 recruit*.

g *Cumulative - includes those with DSA = 0*.

h *Scored as C4d PTC*.

i *μmol/L*.

j *mls/min/1.73 m^2^*.

k *mg/mmol*.

ø *P, NS*.

† *P ≤ 0.005*.

* *P < 0.05*.

*1° renal diagnosis: DM, diabetes mellitus; APKD, adult polycystic kidney disease; GN, glomerulonephritis; SLE, systemic lupus erythematosus; HT, hypertension; TIN, tubulointerstitial nephritis; HUS, haemolytic uraemic syndrome; CNI, calcineurin inhibitor; Tx type: LURD, living unrelated donor; LRD, living-related donor; HLA MM: Number of Class I (A,B) and Class II (DR) mismatches. Two patients (B003 and W007) had their transplants abroad and tissue typing was unavailable. HLA Ab status: CRF, calculated reaction frequency; DSA, donor specific antibody; MFI, cumulative median fluorescence intensity. Means are shown for whole group, including those with MFI of 0. Enrolment biopsy scores: g, glomerular inflammation; ptc, peritubular capillary inflammation; c, chronic scores; TA/IF, tubular atrophy/interstital fibrosis; Immunosuppression: Tac, Tacrolimus; CsA, ciclosporin; MPA, Mycophenolic acid or mycophenolate mofetil. Baseline renal function: eGFR, estimated glomerular filtration rate (4 value MDRD); Formally deteriorating, meet criteria for deteriorating function based on analysis of 1/creatinine plot; Medication: ACE-I, angiotensin converting enzyme inhibitor: ARB, angiotensin II receptor blocker*.

### Results of the RCT

Since the RCT element of the trial was found to be significantly underpowered, and recruitment was halted prematurely, detailed descriptions of the per-protocol RCT population, and the results of the primary and secondary endpoint analyses not described here, are contained within the Supplementary Text, Supplementary Tables 4, 5, and Supplementary Figures 3, 4. There were no significant differences in any measured primary or secondary outcomes between the 11 controls and 9 rituximab-treated patients at the planned second interim analysis and beyond.

### Associations and Outcomes in Patients After IS Optimization

**Figure 2:**
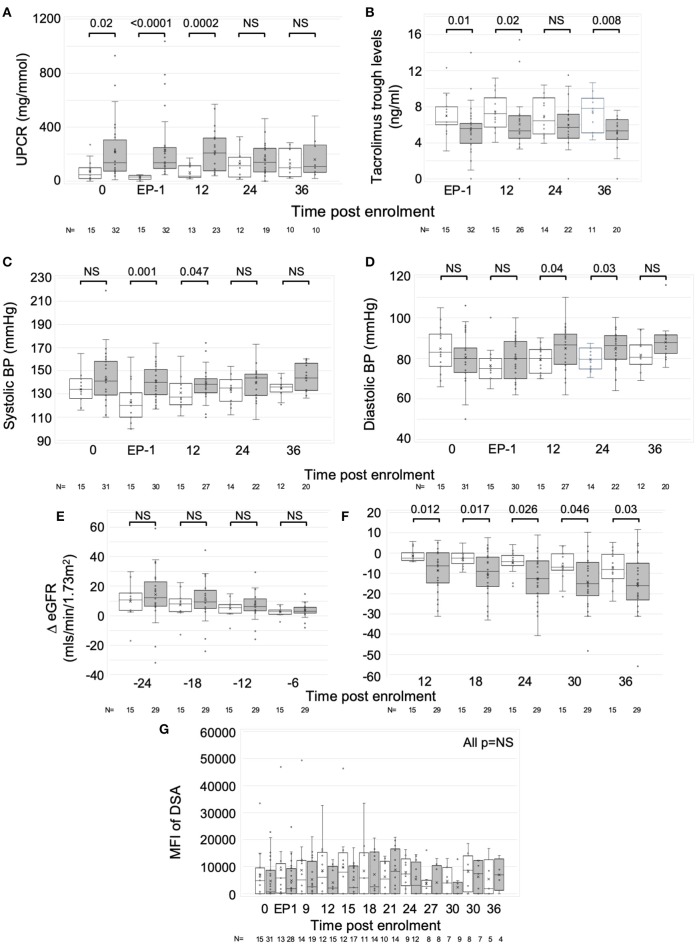
Response to optimized immunosuppression. Exploratory analysis comparing those who responded favorably to optimized IS with those who did not. Graphs are box and whisker plots showing median with interquartile range (IQR) with whiskers showing upper and lower limits and outliers indicated as single data points. Means are represented with “x.” Time points: 0, enrolment sample; EP-1, End phase 1; 0–36, months post enrolment. White bars (*n* = 15); patients who responded well to optimized IS. Gray bars (*n* = 31 pre-enrolment. *n* = 32 post (one recruit did not have sufficient pre-enrolment creatinines); patients who failed to respond to optimized IS. **(A)** Urine PCR, **(B)** Tacrolimus trough levels, **(C,D)** Systolic, **(C)** and diastolic, **(D)** blood pressure (BP), **(E,F)** ΔeGFR normalized to enrolment ΔeGFR of 0. **(G)** Changes in Median Fluorescence Intensity (MFI) of cumulative DSA with time (NB includes values where DSA = 0). *P*-values by Mann Whitney *U*-test.

The group responding poorly to optimized IS (*n* = 32) were well matched in age, sex, ethnicity and multiple other baseline characteristics, including recruitment biopsy features, baseline immunosuppression and proportion with DSA and DSA MFI, to those who responded favorably (*n* = 15) (Table 1). However, more of poor responders had a baseline PCR >50 mg/mmol and the mean PCR was significantly higher (213 ± 211 vs. 74 ± 74; Table 1, Figure 2A). Post-optimization, there were equal proportions established on tacrolimus, MPA, ACE-I and ARB (Table 1), but the good responders had higher levels of tacrolimus (Figure 2B) and better BPs (Figures 2C,D). Importantly, the good responders maintained a significantly lower ΔeGFR over time and had lower levels of proteinuria for 12 months compared to those responding poorly to optimized IS (Figures 2E,F) There was a non-significant trend toward lower graft failure rates (3/15 vs. 12/32), but no differences in DSA MFI in the two groups over time (Supplementary Table 6, Figure 2G), nor differences in adverse event rates (Supplementary Table 5).

### Changes in Circulating B Cells

**Figure 3:**
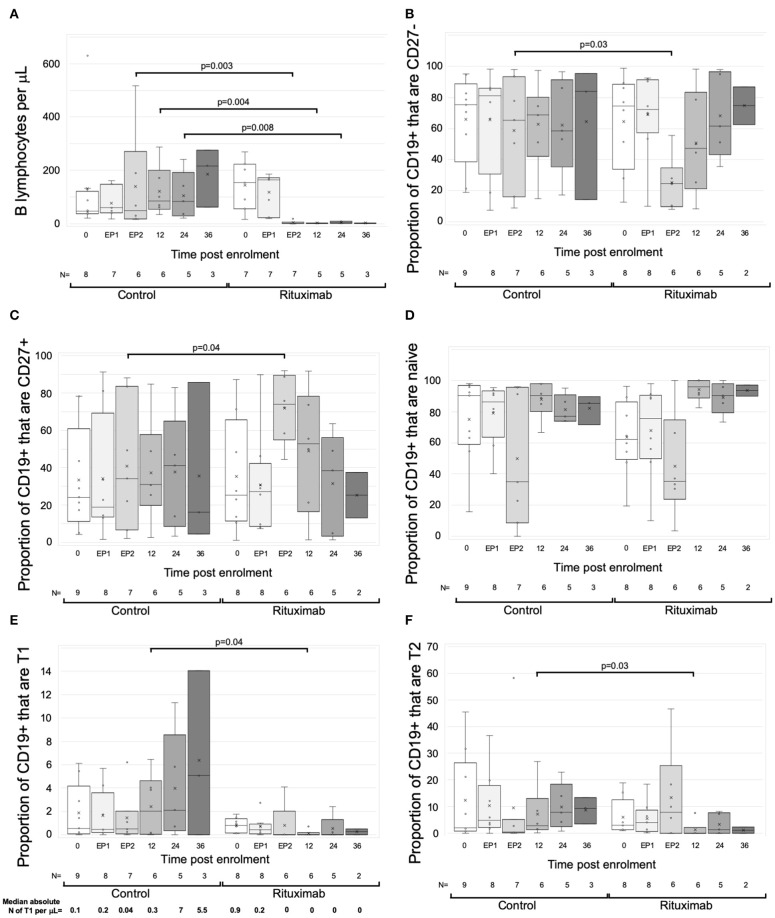
Changes in B cells with rituximab—data from RCT per protocol groups. Graphs are box and whisker plots showing median with interquartile range (IQR) with whiskers showing upper and lower limits and outliers indicated as single data points. Means are represented with “x.” Time points: 0= enrolment sample. EP-1, End phase 1; EP-2, End phase 2; 0–36, months post enrolment. Rituximab administered between EP-1 and EP-2. The gating strategy is described in detail in methods. “N” refers to the number of samples at each time point. **(A–F)** Changes in B cells in RCT. **(A)** Absolute numbers of B cells per uL of serum. **(B–F)** Flow cytometric analysis of the proportion of B cell subpopulations against time. **(B)** CD27-negative B cells as proportion of total CD19+ cells. **(C)** CD27+ B cells as proportion of CD19+ cells. **(D)** CD38loCD24lo cells as proportion of CD27- cells (naïve B cells). **(E)** CD38++CD24++cells as proportion of CD27- cells (Transitional T1 cells). Median absolute number of T1 per μL is shown beneath each column. **(F)** CD38+CD324+ cells as proportion of CD27- cells (Transitional T2 cells). *P*-values by Mann Whitney *U*-test.

Circulating B cells were assessed in real time from eight RCT control and seven rituximab-treated patients from 2 centers. At enrolment and end phase 1, B cell numbers were similar. Beyond phase 2, the rituximab-treated group had significantly fewer B cells to the end of year 3, with a median reduction of 98.2% (IQR 3.8%), though sample numbers at the year 3 time point were too small to make statistical comparisons (Figure 3A).

Exploratory analytical samples from nine control and eight rituximab-treated patients were analyzed as described above and previously (13, 14). Immediately post-rituximab, the B cells remaining were transiently skewed toward a memory (CD27+) phenotype (Figures 3B,C). Within the CD27- population, relative proportions of naive cells were similar, but transitional cell subpopulations T1 and T2 were reduced in rituximab-treated patients at all time points (Figures 3D–F), though statistically significant differences were only seen at end of year 1. Median absolute numbers of T1 cells were low (<1 cell per μL) at enrolment, consistent with previous reports (28). However, whilst numbers of T1 cells appeared to increase in the control population, they were undetectable post-rituximab (Figure 3E). These trends were also evident when rituximab-treated patients were compared to all non-rituximab-treated controls included in the exploratory analysis (Supplementary Figure 2).

### Association Between Specific Subsets of CD4+ and CD19+ Cells and Patterns of Indirect Pathway Anti-donor IFNγ Production

**Figure 4:**
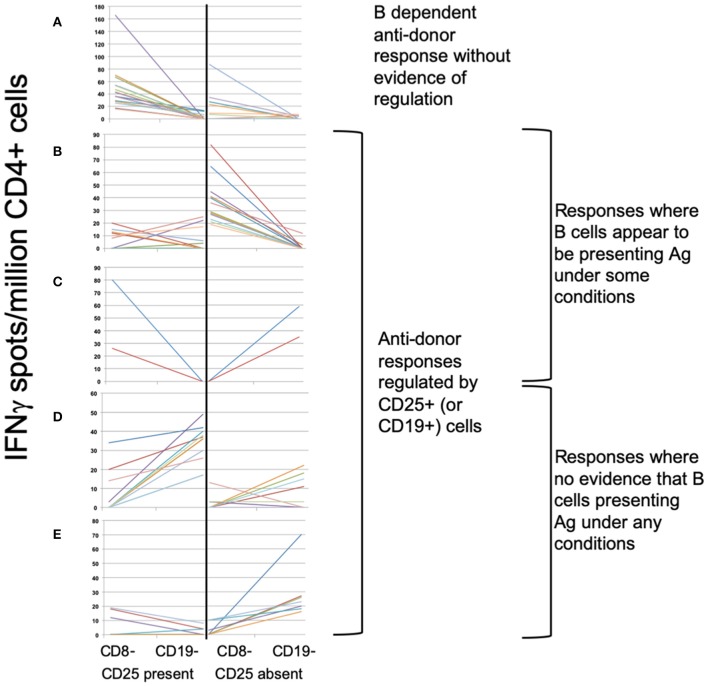
ELISPOT patterns. **(A–E)** Illustrates the 3 basic patterns of anti-donor IFNγ production, displayed as the spot count (corrected for flow cytometric assessment of CD4+ cell proportions) present under 4 different conditions: CD8- (CD8-depleted PBMC); CD19- (CD8- & CD19-depleted PBMC), both performed in presence or absence of CD25+ cells. Samples showing anti-donor responsiveness from 51 recruits, including from those not in the exploratory analysis, are represented. **(A)** Pattern 1: Unregulated B cell-dependent pattern. Showing spot counts that reduce (>20%) on depletion of CD19+ cells in presence of CD25+ cells (± in absence of CD25+ cells). *N* = 16 samples. **(B,C)** Pattern 2: B cell-dependent anti-donor patterns with evidence of regulation^1^. **(B)** CD25+ regulated B-dependent responses: B cell-dependent anti-donor responses only detectable in absence of CD25+ cells. *N* = 14 samples. **(C)** CD19+ regulated B-dependent responses. B cell-dependent anti-donor responses in presence of CD25+ cells, but when CD25+ cells absent, depletion of CD19+ cells increases spot count (>20%), indicating evidence of regulation by B cells. *N* = 2 samples. **(D,E)** Pattern 3: Regulated anti-donor responses without evidence of B cell-dependency. **(D)** CD19+ regulated responses; In presence of CD25+ cells, spot counts increase (>20%) when CD19+ cells are depleted. *N* = 11 samples. **(E)** CD25+ and CD19+ regulated. In absence of CD25+ cells, depletion of CD19+ cells increases spot counts (>20%). In presence of CD25+ cells, anti-donor responses are undetectable. *N* = 7 samples.

**Figure 5:**
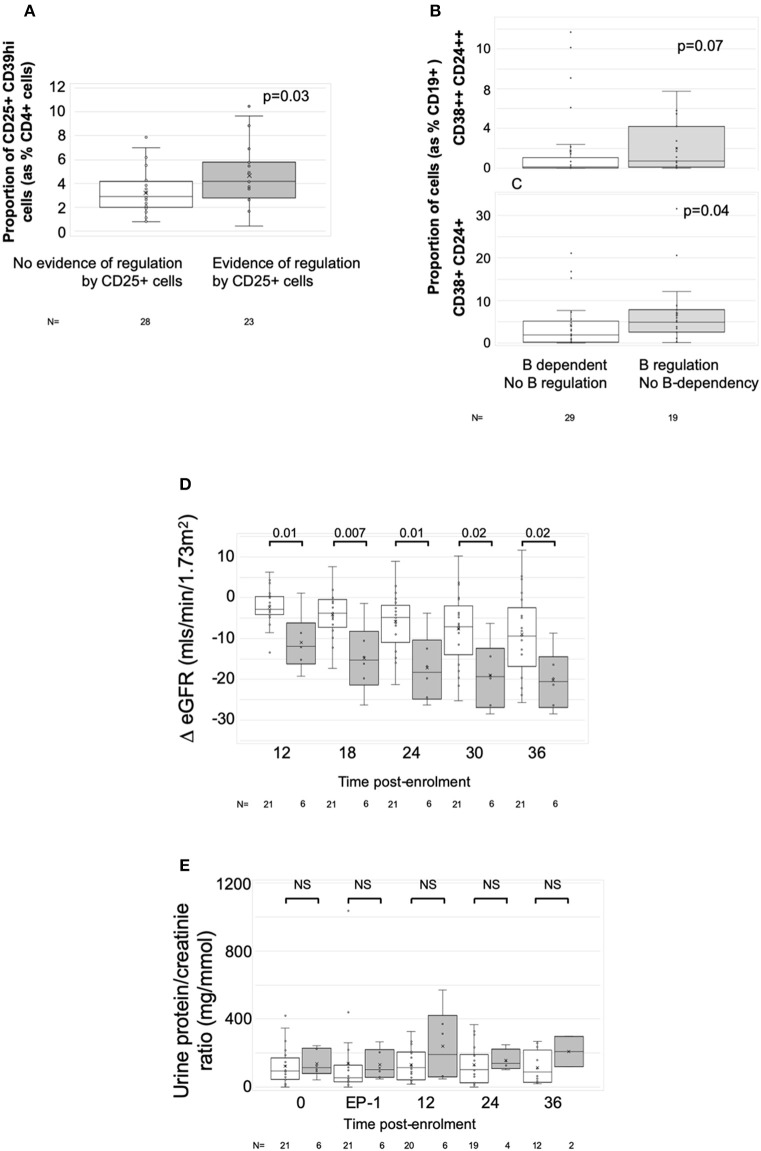
Associations with ELISPOT patterns. Graphs show box plots of median with IQR with whiskers showing upper and lower limits and outliers indicated as single data points. Means are represented with “x.” **(A)** Association between proportion of CD4+CD25+CD39hi T cells (Tregs) and ELISPOT patterns characterized by spot count suppression when CD25+ cells present. **(B,C)** Association between proportion of CD19+ cells having the phenotype of transitional T1 cells (CD27-CD38++CD24++) **(B)** or transitional T2 cells (CD27-CD38+CD24+) **(C)** and ELISPOT patterns showing evidence of increasing spot counts after depletion of CD19+ cells. **(D,E)** ΔeGFR, normalized to enrolment eGFR of 0 **(D)** and urine PCR **(E)** in patients with at least two samples at end-phase 2 or beyond (*n* = 27). White bars are patients who had either donor non-responsiveness or ELISPOT patterns with evidence of regulated anti-donor responses in their post-optimization samples (*n* = 21). Gray bars are those with at least one post-end-phase 2 sample showing evidence of unregulated B cell dependent anti-donor responses (*n* = 6). Time points: 0, enrolment sample; EP-1, End phase 1; EP-2, End phase 2; 0–36, months post enrolment. *P*-values by Mann Whitney *U*-test.

To define associations between cell phenotype and anti-donor ELISPOT patterns, 203 samples from 51 recruits were analyzed, including from 4 recruits not entered in the main exploratory analysis. In these assays, PBMC were depleted of CD8+ (cytotoxic) T cells, before sequential depletion of CD25+ (predominantly regulatory) T cells and CD19+ (all B) cells, to assess the roles that these cell types played in the response. Figure 4 illustrates the patterns seen in these samples and how we defined them. 58/203 showed an IFNγ response to donor antigens indicating the presence of specific anti-donor CD4+ T cells. In 30/58 samples, B cell depletion reduced the number of responding spots, which, as previously shown (13), suggests that B cells were presenting donor antigens. In 14 of these 30 B-dependent samples, there was an increase in the number of responding spots when CD25+ cells were depleted, indicating that regulatory T cells were suppressing these B-dependent responses. Samples displaying this pattern of regulation contained a significantly higher proportion of CD4+CD25+CD39hi regulatory T cells, compared to other reactive samples (Figure 5A). In 20/58 further samples, the number of responding spots increased when B cells were depleted, suggesting that B cells were actually suppressing anti-donor CD4+ T cells. In 9/20 of these samples showing apparent regulation by B cells, this type of response was only evident when CD25+ cells were depleted; spot counts in 7/9 of these were completely suppressed when CD25+ cells were present. In the remaining 2/9 samples, the response was B-dependent when CD25+ cells were present, implying complex interactions between CD25+, CD19+ and donor reactive T cells. Nevertheless, all 20 samples where B cells appeared to be suppressing IFNγ production contained a higher proportion of T1 (CD38++CD24++) or T2 (CD38+CD24+) transitional B cells (Figures 5B,C). The final 8/58 samples had patterns of B-dependent responsiveness that differed depending on the dose of antigen used, making it difficult to associate patterns of responsiveness with cell phenotype.

### Changes in Anti-donor IFNγ Production After Optimization of IS vs. Post-rituximab

Only samples from the patients included in the exploratory analysis who had enrolment PBMC collected (*n* = 43) were included in this analysis, and the patterns from the enrolment samples were compared to 128 samples taken from the same patients after optimization of immunosuppression (Table 2 and Supplementary Tables 7, 8). The proportion showing either no anti-donor responses, or responses regulated by CD25+ or CD19+ cells increased, whereas those showing B-dependent responses without any evidence of regulation reduced (Table 2). These data suggest that optimization of IS inhibited unregulated B-dependent responses but promoted non-responsiveness or regulation.

**Table 2:** IFNγ production patterns in ELISPOTs of 171 samples from 43 patients in the exploratory analysis, comparing patterns at enrolment, with those following optimization.

**Interpretation based on pattern of anti-donor responsiveness**	**Number of samples (%)**		
	Enrolment (*n* = 43)*	**Post-optimization (*n* = 128)**	**Total (*n* = 171)**
No response	26 (60.5%)	89 (69.5%)	115 (67.3%)
CD25+ or CD19+ regulated	8 (18.6%)	29 (22.6%)	37 (21.6%)
B-dependent—no regulation	8 (18.6%)	8 (6.3%)	16 (9.3%)
Not viable/Not interpretable	1 (2.39%)	2 (1.6%)	3 (1.8%)

* *This analysis is of samples from 43 of the 47 recruits included in the exploratory analysis, who had enrolment PBMC collected*.

*Comparing enrolment vs. post-optimization patterns in a Fisher 2 × 3 exact probability test [(No response + regulated response) vs. B-dependent responses vs. non-viable], p = 0.04*.

*Refer to Supplementary Tables 7, 8 to see the individual ELISPOTs from all 43 patients included in this table*.

To examine the impact of rituximab, ELISPOT patterns from the same patients were reorganized to compare pre- and post-phase 2 patterns in patients who received rituximab to those not receiving it (including samples from outwith RCT controls) (Table 3). Comparison of only those samples showing anti-donor responses revealed that post rituximab there was a reduction in the proportion of donor-reactive responses suppressed by CD25+ or CD19+ cells (such that none of the post-phase 2 donor-reactive samples from the rituximab group showed suppression by CD19+ cells and only 2 showed suppression by CD25+ cells see Supplementary Table 7), whilst the proportion of samples showing unregulated B-dependent IFNγ production increased (Table 3). These changes just failed to reach statistical significance. Together these data suggest that rituximab opposed the impact of optimized IS, by inhibiting regulated anti-donor responses, particularly by B cells, but failing to inhibit non-regulated B-dependent responses.

**Table 3:** Anti-donor IFNγ production patterns in ELISPOT arranged to illustrate the effect of rituximab.

		**Exploratory group**	
		**No rituximab**	**Rituximab**
Before end phase 2	CD25+ or CD19+ regulated anti-donor response	9	8
	B-dependent anti-donor response – no regulation	7	3
End phase 2 and beyond*	CD25+ or CD19+ regulated anti-donor response	18	2
	B-dependent anti-donor response – no regulation	3	3

*Samples from the same population as that represented in Table 2, re-organized according to whether patients received rituximab or not*.

*Differences between groups compared by Fisher 2 × 2 Exact probability test*.

*Comparing Elispot patterns in pre-phase 2 samples from those not receiving rituximab with those receiving rituximab; p = 0.44. In contrast, comparing patterns in post phase 2 samples from those not receiving rituximab with those receiving rituximab; p = 0.06*.

* *i.e., post-rituximab in rituximab-treated patients*.

*Refer to Supplementary Tables 7, 8 to see the individual ELISPOTs from all 43 patients included in this table*.

### Association Between Changes in Anti-donor IFNγ Production and Kidney Function

Twenty-seven patients had sufficient numbers of viable and interpretable ELISPOTs for analysis of dynamic change (Supplementary Table 7 and main Table 4). Irrespective of the enrolment ELISPOT, the presence of a single non-regulated B-dependent response any time beyond end of phase 2 in 6 patients was associated with a statistically significant greater fall in ΔeGFR at 3 years compared to 21 patients who remained non-responsive or had evidence of regulated anti-donor responses (Table 4). The difference in ΔeGFR became evident by 12 months after enrolment (Figure 5D). There was no association with PCR (Figure 5E). 9 of the 15 patients who responded well to optimized immunosuppression had enough ELISPOT s to be included in this analysis and none had evidence of unregulated B-dependent anti-donor responses beyond end of phase 2 (Supplementary Table 7). In contrast, of the 18/32 patients who responded poorly to optimized IS with enough samples to be included, 6 had evidence of non-regulated B-dependent responses beyond the end of phase 2 (Supplementary Table 7). This included 3 of 7 who received rituximab, 1 of 6 RCT controls, and 2 of 5 patients who responded poorly to optimized IS but were not randomized. These data support the conclusions made from a previous observational cohort (13, 14) and suggest that the presence of non-regulated, B-cell–dependent anti-donor IFNγ production after treatment with either optimized IS or rituximab is associated with a risk of significant decline in eGFR.

**Table 4:** Dynamic changes in ELISPOT patterns in twenty seven patients with viable and interpretable enrolment samples and at least two viable and interpretable samples at or beyond end of phase 2.

		**ELISPOT patterns at or beyond end phase 2**	
		No response OR CD25+/CD19+ regulated anti-donor response	B-dependent anti-donor response—no evidence of regulation*
Pre-end phase 2	Any ELISPOT pattern	*N* = 21 (4)^†^ Median ΔeGFR−9.41 (IQR 9.5)^∅^	*N* = 6 (3) Median ΔeGFR−20.55 (IQR 7.8)^ø^

* *Only includes patients with enrolment and 2 or more viable and interpretable post treatment ELISPOTs. If any of these ELISPOTs showed evidence of non-regulated B-dependent anti-donor IFNγ production, the patient is included in one of these two columns*.

† *Numbers in parentheses indicate the number with failed grafts or withdrawals due to IS reduction as a prelude to starting dialysis*.

ø *Comparison of median ΔeGFR at 3 years: p = 0.02 by Mann Whittney U*.

*Refer to Supplementary Table 7 to see the individual ELISPOTs from all 27 patients included in this table*.

## Discussion

Immune mediated injury is now recognized as an important cause of late allograft dysfunction and failure after kidney transplantation (4, 34, 35), though there are still no widely available or established treatments. The RituxiCAN-C4 trial addressed whether rituximab could stabilize graft function in patients already optimized on oral immunosuppression. This investigator led RCT was terminated prematurely when it became clear, after a planned interim analysis, that it had been significantly underpowered. Redesigning the trial, based on observed effect sizes was considered unfeasible, because the slow recruitment encountered had meant that funds to continue the trial had been used up. Nevertheless, after formal analysis of all endpoints, we found no evidence that depleting B cells after a failed trial of optimized IS could stabilize kidney function or reduce proteinuria.

Since RituxiCAN-C4 started, multiple other groups have described the use of Rituximab in patients with CAMR. Seven retrospective cohort studies between 2012 and 2016 were summarized in a recent systematic review by Macklin et al. (36), who noted that one reported a short-term stabilization in eGFR and better graft survival benefit, three reported no difference and three others reported worse outcomes associated with Rituximab. A meta-analysis was not possible because of the heterogeneity of inclusion criteria and treatment protocols, most of which also included IVIg and plasma exchange. Since then, two other retrospective cohort studies in patients with CAMR have reported. Pineiro et al. reported no difference in graft survival, proteinuria, eGFR and HLA antibodies between 23 patients treated with a combination of Rituximab, plasma exchange and IVIg and 39 untreated controls, but did report an increase in infections (37). Using a similar treatment protocol, Mella et al. reported improvement in biopsy appearances but no differences in allograft survival, allograft function or DSA in 9 treated patients compared to 12 controls (38). Most recently, Moreso et al. reported the results from Triton, the first double-blind phase IIb RCT in patients with CAMR, showing no improvement in eGFR at 12 months in 11 treated vs. 12 controls, and no impact of rituximab on DSA, proteinuria or adverse events (39). RituxiCAN-C4, a similar sized RCT to Triton, has yielded entirely consistent results.

In our previous observational study, we reported that optimization of oral immunotherapy, based around achieving highest tolerated MPA dose and target trough levels of tacrolimus of 4–8 μg/ml could stabilize graft function in patients with CAMR (14), in agreement with earlier reports (40). Therefore, the first phase of RituxiCAN-C4 involved establishing all patients on these drugs, and eligibility for the RCT was assessed once the response to optimization had been considered. This complicated the design, but also offered an opportunity to perform an exploratory observational study in all recruited patients, to study the activity of cell mediated anti-donor responses, determine whether these associated with outcome, and determine how these were influenced by different treatments. Optimized therapy was defined according to what each patient could tolerate, so patients remained enrolled even if they could not tolerate all aspects of the optimized therapy. Although Moreso et al. also included a first phase during which patients were switched to tacrolimus and MPA before randomization, they did not report the impact of this maneuver. We can conclude that 25% of recruits responded favorably to optimized IS. These had similar baseline kidney function and eGFR decline before enrolment as non-responders, but a greater proportion had a PCR <50 and average PCRs were significantly lower. The response to IS optimization in this group was sustained and associated with higher trough tacrolimus levels and better BP control than seen in non-responders; unfortunately, our data does not allow further interpretation of the interplay between these factors.

Rituximab had a definite measurable biological effect; it depleted >95% of circulating B cells for a prolonged period. Our data on repletion of all B cells is consistent with what has been reported in adult renal transplant recipients given a single dose of Rituximab at the time of transplantation. In this group, recovery of peripheral B cell numbers begins around 15 months (21), with <10% recovery by 24 months (22) and ~30% recovery by 36 months (21).

However, our analysis of how B cell subpopulations repopulated after rituximab is different to what's been reported before. We revealed a significant and sustained reduction in the proportion of transitional cells, as detected by expression of CD24 and CD38, whilst the proportions of memory and naïve subsets quickly normalized to those seen in controls. Previous reports have suggested preferential recovery of transitional B cells, with some also describing a sustained reduction in the proportion of memory cells. For instance, in a pediatric population, Zarkin et al. used 4 doses of Rituximab (375 mg/m2) with pulsed steroids to treat acute cellular rejection, showing that 12 months later [by which time peripheral B cell numbers have returned to normal in children (41)], there was a selective expansion of naive B cells, and a reduction in the proportions of memory B cells, compared to a control group treated with steroids alone (23). Sidner et al. treated 9 adult dialysis patients with a single dose of Rituximab, up to 375 mg/m2, and followed reconstitution for up to 2 years (24). Whilst some subsets recovered fully by 6 months, the proportion of CD27+ memory B cells remained low for up to 2 years. Kamburova et al. studied 12 patients receiving single dose Rituximab (375 mg/m2) at the time of transplant. Within the still-depleted peripheral B cell population at 24 months, there was a significant over representation of both transitional B cells (and also switched memory cells) with reduced proportions of naïve cells (22). Two other groups, using low dose Rituximab (200–800 mg) as induction therapy or as adjunct therapy for acute rejection reported preferential expansion of transitional cells with a reduction in the proportion of memory B cells, 3–10 months post-treatment (25, 26). Therefore, the pattern of B subset repopulation we describe in this cohort is distinct from those described in these other transplant cohorts.

Our exploratory analyses of IFNγ production suggested a functional impact of both optimizing immunosuppression and rituximab on anti-donor alloresponses. By adding whole antigens into CD8-depleted PBMC, the ELISPOT assays measure the activity of the indirect pathway of allorecognition (42), and we used changes seen when CD25+ or CD19+ cells were depleted to infer the functional impact of these cells on anti-donor responses. Where possible, we used donor antigens isolated from cells obtained either at the time of transplantation or from new samples donated by a living donor. For those where no donor material was available, we used surrogate donor cells. Whilst these surrogates were chosen on the basis of a hierarchy of rules (listed above), designed to minimize the possibility of measuring irrelevant indirect alloresponses, it is impossible for us to be certain that we have eradicated this risk, and our data has to be interpreted with this in mind.

Our results imply a level of complexity as previously described in another cohort (13), which we have tried to simplify here by describing three broad patterns: non-responsiveness, regulated anti-donor IFNγ production, or B dependent anti-donor responses with no evidence of regulation. These categorizations are useful as they associated with clinical outcomes in a previous cohort (14). B cell dependent responses [previously shown to indicate that B cells are presenting donor antigen to CD4+ T cells (13)], were found in 20% of samples, but in 12% of these, anti-donor reactivity was only revealed by depletion of CD25+ or CD19+ cells, indicating functional regulation of IFNγ production by these cells. A further 9% showed responses in which B cells acted purely to suppress CD4+ T cell responses. Regulation of IFNγ production by CD25+ T cells associated with a higher proportion of CD4+CD25+CD39hi cells, a phenotype consistent with Tregs (43), whereas regulation by CD19+ B cells associated with higher proportions of transitional B cells, a population with a known regulatory phenotype.

These phenotypes appear dynamic, with some patients demonstrating all three broad patterns of ELISPOT reactivity over the course of the study. Although we have not specifically addressed why anti-donor responses detectable in PBMC change over time in some individuals, our data suggests that the changes relate to the relative proportions of CD25+ and transitional B cells present in the peripheral blood at any particular time. We think these dynamic changes in anti-donor IFNγ production have biological significance, because as we have previously reported, they associate with change in eGFR (14). Six patients had an unregulated B-dependent pattern in at least one of their samples in the follow-up period, and these had a significantly greater deterioration in eGFR, compared to others. Three of these six received rituximab. No rituximab-treated patients showed evidence of CD19+ suppressed anti-donor responses post-rituximab. Interpreted alongside the impact of rituximab on the relative proportions of transitional and memory B cell subsets, these data suggest that rituximab depleted regulatory B cells, but not those presenting donor antigen to T cells. These unique and novel observations, albeit in small numbers of patients, provide a potential explanation for why rituximab lacks efficacy, consistent with that proposed when rituximab, used as an induction agent, appeared to cause a significant increase in acute rejection (44).

We acknowledge that there are several serious shortcomings of this study. First, our biopsy inclusion criteria diverge from the widely used BANFF classification of kidney allograft pathology. From the beginning, we chose to include DSA-negative recruits and placed as much emphasis on the presence of glomerular C4d (by IHC) as PTC C4d. However, considering that DSA-neg CAMR is reported to have the same natural history and prognosis as DSA+ CAMR (45) and the diagnostic significance of glomerular C4d by IHC is now appreciated (46–48), we consider that the inclusion of these patients is justified. In addition, significant revisions of BANFF criteria have occurred since we started, most importantly removing the presence of TA/IF as evidence of damage associated with DSA (from BANFF 2013 onwards). However, the last patient in the randomized population (i.e., included in the primary endpoint analysis) was recruited on a biopsy taken in November 2013, before the formal publication of the BANFF 2013 criteria, so it would have been impossible to alter recruitment to the RCT based on BANFF 2013. Nevertheless, all biopsies have been re-examined for this report, and 17/20 patients included in the primary EP analysis had biopsies that met histological criteria for CAMR by BANFF 2013 (Table 2). Of the remaining 3 not meeting these criteria, 2 lack TG or PTCBMML, but have significant TA/IF and 1 has TG but lacks both PTC C4d and microvascular inflammation scores ≥2. With regard to the exploratory analysis, 41/47 had enrolment biopsies that fulfill the BANFF 2013 histological criteria for chronic active AMR. As well as the 3 biopsies already mentioned, 1 additional biopsy lacks TG or PTCBMML but has TA/IF and 2 others have evidence of TG or PTCBMML but lack PTC C4d or microvascular inflammation scores ≥2. Thus, since 85% of patients included in the RCT and 87% of those in the exploratory analysis meet present day histological criteria for chronic active AMR, we propose that our conclusions are still relevant to a modern transplant population.

Second, we've reported that all potentially eligible patients identified from review of allograft biopsies consented to enter phase 1. However, we did not collect data on the proportion of reviewed biopsies meeting eligibility criteria, which is a potential flaw. In addition, we didn't mandate that centers adopt uniform criteria for performing biopsies (this was a clinical decision), and the majority of patients were recruited from 2 centers, so we cannot exclude selection nor center bias in these data.

Third, our anticipated effect and sample sizes were based on a small non-randomized internal pilot (49), but this overestimated the benefits of rituximab, as revealed by the second interim analysis, so the trial was significantly underpowered. These problems were compounded by the fact that recruitment was very slow (such that funding had expired) and this impacted on the decision to halt recruitment rather than re-power the trial.

Fourth, our exploratory data is also derived from small numbers of patients, particularly those from the rituximab-treated group, and as grafts failed or patients withdrew, sample numbers from the later time points in phase 3 dropped, making statistical comparisons between groups at these later time points difficult.

Nevertheless, the data supports previously reported findings, providing further evidence of a link between anti-donor IFNγ production and progressive loss of eGFR in patients with CAMR and suggesting that B cells appear to play a complex and dynamic role in either supporting or regulating IFNγ production. Whilst optimization of oral IS appears to suppress anti-donor IFNγ production and associates with a sustained improvement in eGFR in some patients, rituximab appears to disturb the balance of the two opposing roles of B cells, by selectively reducing the relative proportion of transitional B cells (associated with regulation), while failing to sustainably deplete B cells that support anti-donor responses, for reasons that are not immediately obvious.

These data suggest that newer anti-B cell therapies, to selectively target B cell subpopulations, or the distinct functions of B cells, may offer a new avenue to treat this difficult clinical problem.

## Data Availability Statement

The data that support the findings of this study are available from the corresponding author upon reasonable request.

## Ethics Statement

The studies involving human participants were reviewed and approved by the West London Committee of the National Research.

## Author's Note

The study was indemnified for negligent and non-negligent harm by King's College London. The corresponding author has had full access to all the data in the study and made the final decision to submit for publication.

## Author Contributions

KS recruited patients, collected and analyzed samples, wrote trial amendments, and edited the paper. DS was the trial statistician and edited the paper. LM, HB, HW, HD, T-LT, and OS analyzed samples in exploratory analysis and edited the paper. OS and PB interpreted the HLA Ab data. AM, RH, SG, CG, SB, and RB recruited PIs, performed data entry, and edited the paper. CR and CH performed histopathology interpretation and edited the paper. AD was CI, designed the study, wrote the protocol, recruited patients, performed data entry, performed analysis of exploratory study, and wrote the paper.

### Conflict of Interest

OS is employed by Viapath Analytics LLP. The remaining authors declare that the research was conducted in the absence of any commercial or financial relationships that could be construed as a potential conflict of interest.

## Footnotes

^1^*NB. 8 samples showed CD25*+ *or CD19*+ *regulated B-dependent patterns that were dependent on dose of antigen and these are not represented here*.
