# Supplementary material for: Effect of Optimized Immunosuppression (Including Rituximab) on Anti-Donor Alloresponses in Patients With Chronically Rejecting Renal Allografts
Source: Front Immunol. 2020 Feb 5;11:79. doi: 10.3389/fimmu.2020.00079 (PMC7012933; doi:10.3389/fimmu.2020.00079)

Supplementary Figure 1

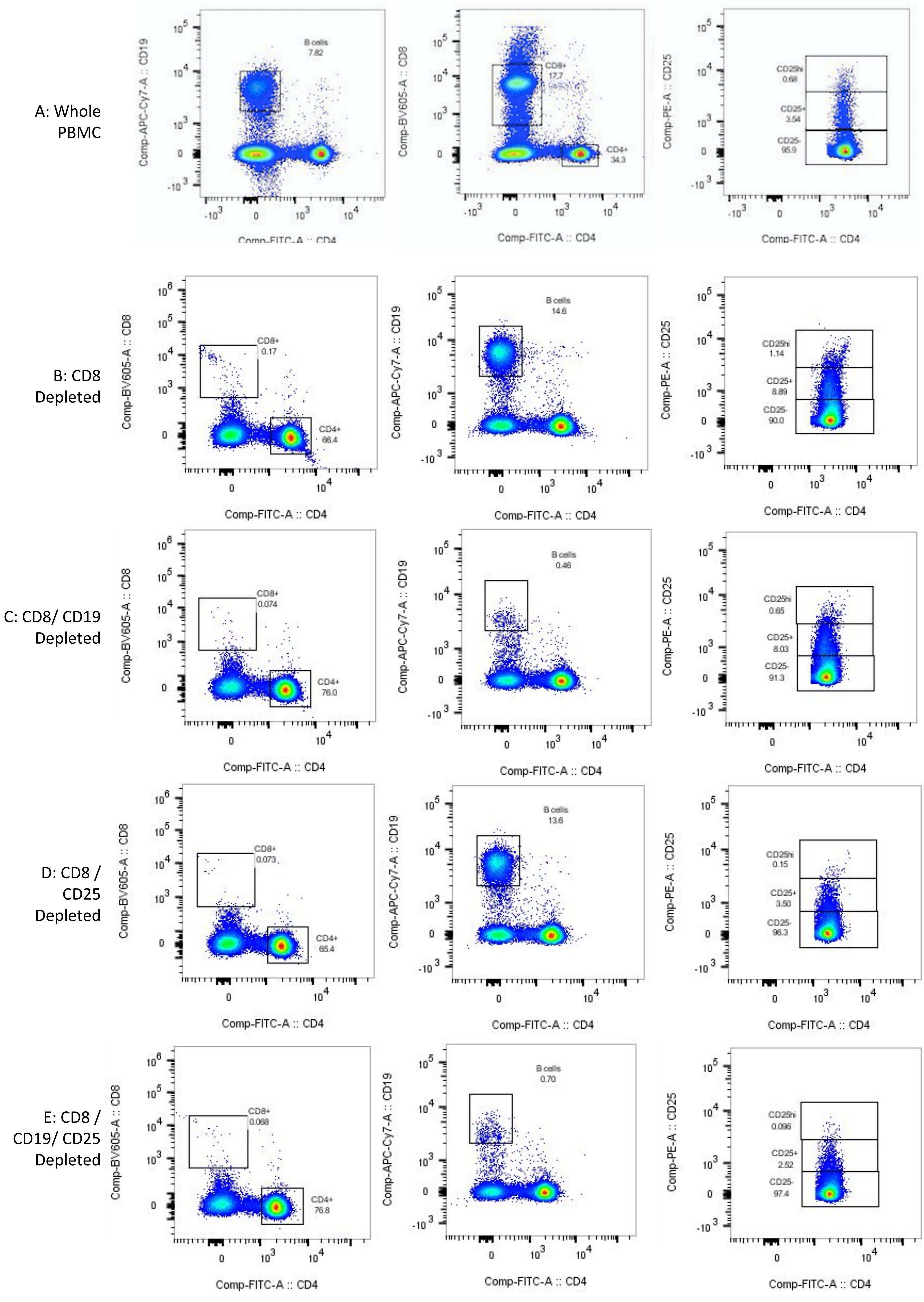

Supplementary Figure 2

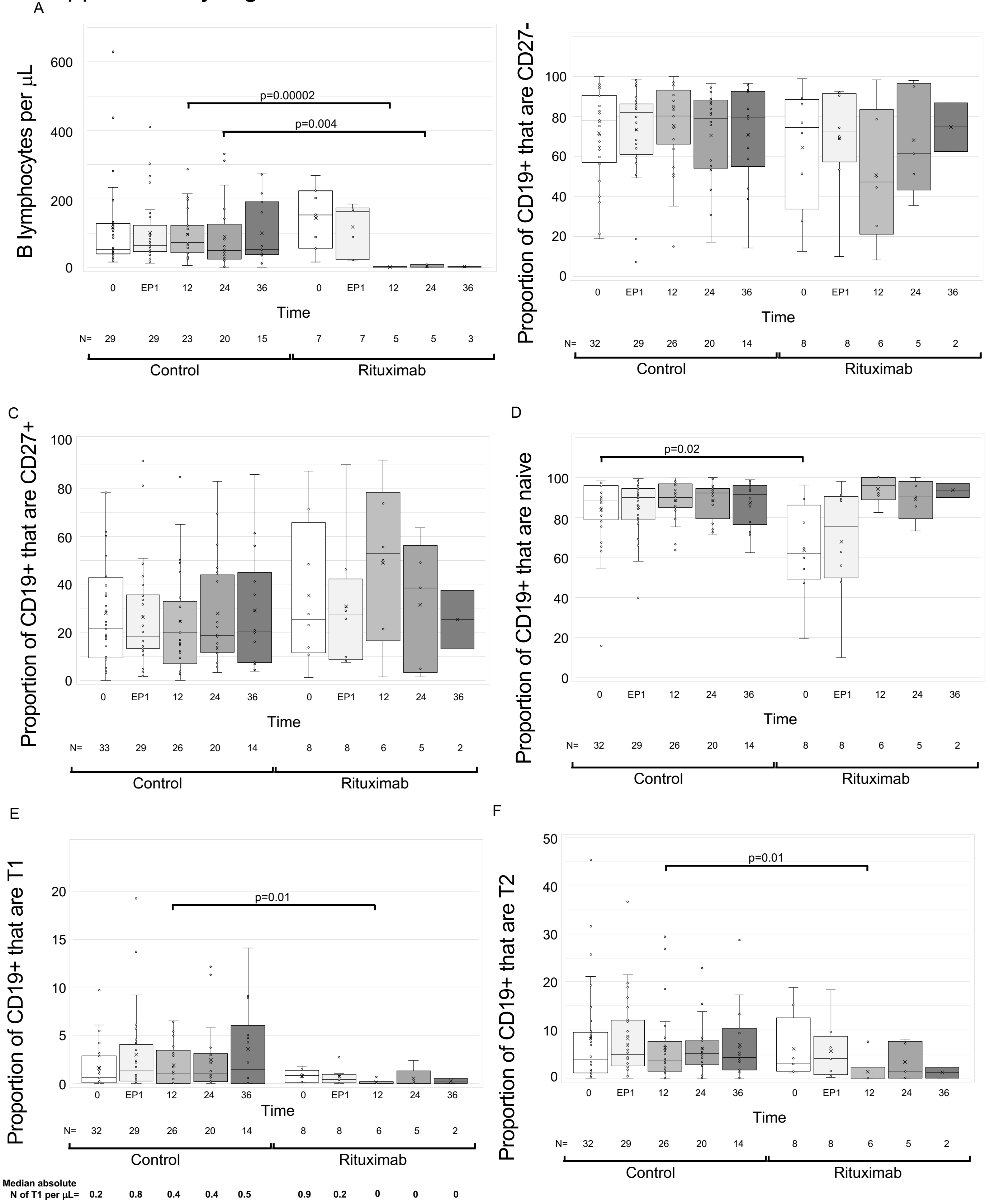

Supplementary figure 3 – 1/creatinine plots from patients included in primary EP analysis of the RCT

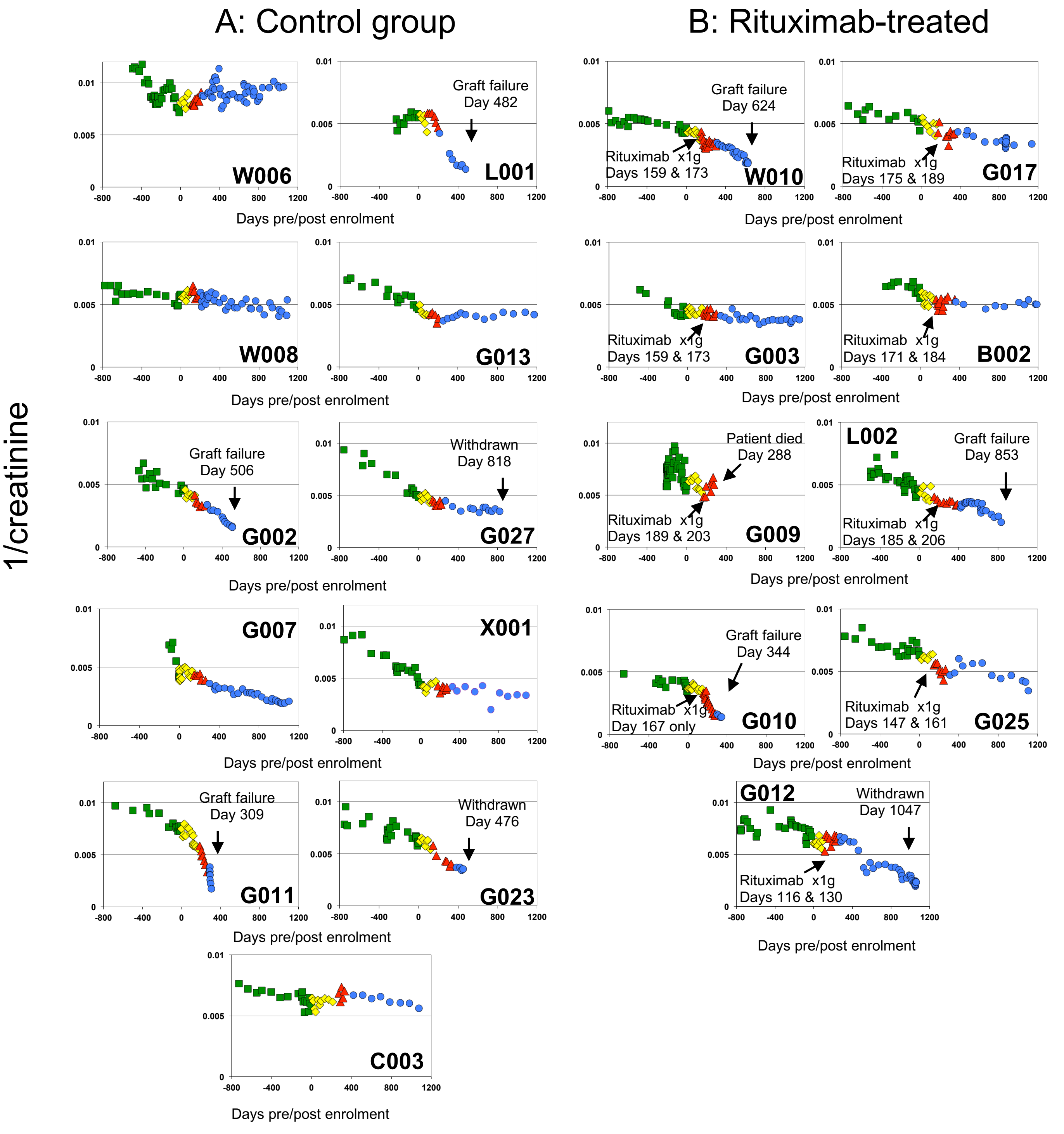

C

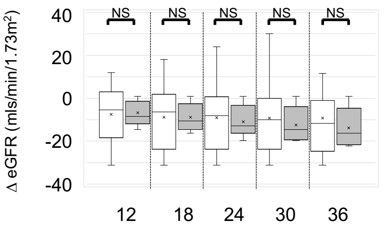

Supplementary Figure 4 – Changes in DSA in patients included in primary EP analysis of the RCT (see supplementary figure 4 for the three patients in the Rituximab-treated group who were not included in in the primary endpoint analysis

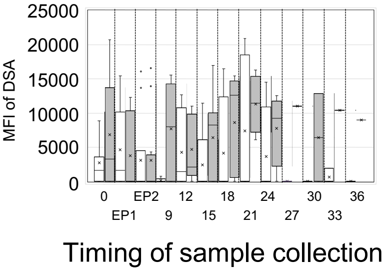

B: Control group

C: Rituximab-treated

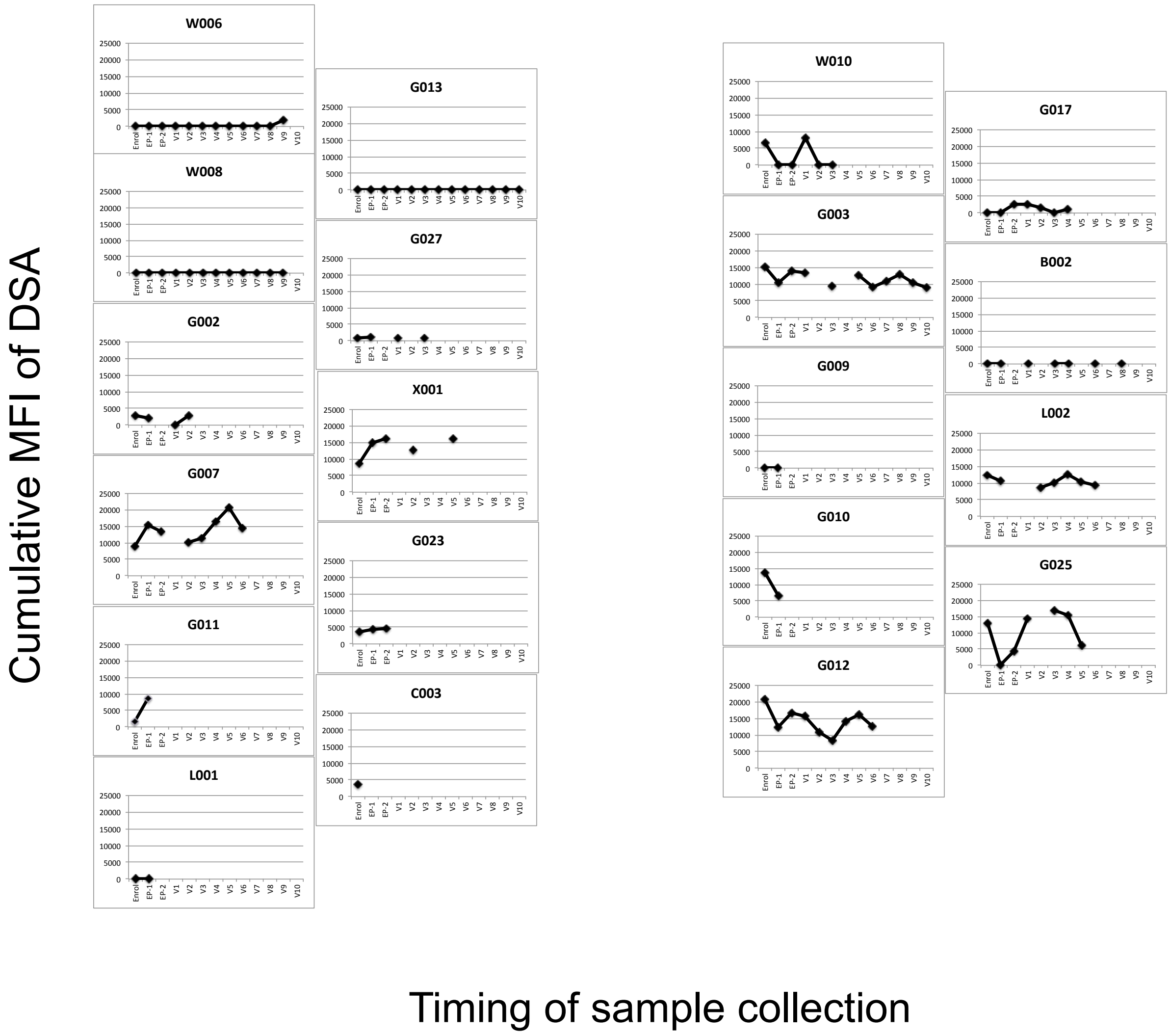

Supplement: Supplementary file 1 [file Data_Sheet_1.PDF]
