# Supplementary material for: Effect of Optimized Immunosuppression (Including Rituximab) on Anti-Donor Alloresponses in Patients With Chronically Rejecting Renal Allografts
Source: Front Immunol. 2020 Feb 5;11:79. doi: 10.3389/fimmu.2020.00079 (PMC7012933; doi:10.3389/fimmu.2020.00079)
Supplement: Supplementary file 2 [file Data_Sheet_2.PDF]

Supplementary Methods:

Procedures (refer to main figure 1)
Optimised therapy was individually tailored and inability to tolerate one or more aspects was not classed as 'failure'. Patients already deemed to be on optimal therapy went straight into a three-month observation period.
At the end of phase 1, patients with an eGFR>20 >20 mL/min/1.73m^2, a PCR ≥50 or continued deterioration of graft function on analysis of a reciprocal creatinine plot , were eligible for the RCT. These patients were asked to consent specifically to randomisation. Patients not meeting criteria and those who refused consent went straight to phase 3, where all protocol-defined interventions ceased, but study observations continued every 3 months for three years post-recruitment. A significant change in IS in any phase was considered a reason for withdrawal. All patients requiring dialysis or re-transplantation were also withdrawn.

Randomisation
Allocation to treatment or control arms was assigned (1:1) by permuted variable block randomisation with random block sizes of 4 or 6. Randomisation was carried out using sequentially numbered opaque envelopes sealed by an independent party in the Pathology Department of Hammersmith Hospital. Block sizes and the allocation schedule were concealed from trial investigators. Following the first interim analysis in July 2011, randomisation was stratified by graft function and proteinuria to ensure equal allocation to each arm, with generation of a new series of envelopes. Envelopes were opened by the CI once consent to the RCT was obtained by the local investigator.

Interventions and blinding
All remained on optimised IS. Controls received no additional treatment. Rituximab treatment consisted of two 1g infusions 14 days apart, administered with paracetamol and chlorpheniramine +/- hydrocortisone, followed by co-trimoxazole (or alternative) for 6 months. Blinding was considered unworkable as circulating B cells were measured as a secondary endpoint (EP).

Primary objective and EP
The primary objective was to determine whether rituximab could stabilise kidney function and/or reduce proteinuria after optimised IS had failed. The primary EP was 3-5 months post-randomisation (dependent on trial arm). There were two co-primary outcome measures:
• Rate of deterioration in graft function, defined by individual slope of the reciprocal creatinine plot (over the preceding 3 months). Slope was assessed continuously, but also as a binary measure, with 'deterioration' defined as a negative individual slope with an adjusted $R^2>0.35$ and $p<0.05$ (1).
• Change in PCR (as continuous measure).

Secondary outcomes
Secondary outcomes, determined 3-5 months post-randomisation and at 1, 2- and 3-years post-recruitment, were: a) deterioration in function (slope of reciprocal creatinine plot since the previous assessment): b) patient and c) graft survival: Incidence of d) infection (with diagnostic certainty) and e) malignancy: Changes in f) PCR, g) anti-graft Ab, h) circulating CD20+ cells and i) T cell responsiveness to donor alloantigens.

Effect size and sample size calculation
A pilot study was performed on 10 consecutive patients between November 2005 and October 2006, all of whom had for-cause biopsies for allograft dysfunction showing CAN by Banff '97 criteria and chronic transplant glomerulopathy associated with diffuse linear C4d staining on > 50% of PTC or glomerular capillary EC (using polyclonal antibody to C4d) (2). Five patients required optimisation of oral therapy and were converted from their baseline drugs onto tacrolimus and MMF. Of these, 3/4 (75%) stabilized within 3 months of optimisation and a fourth stabilized 3-6 months after optimisation. The fifth patient failed to stabilize so received 2 doses of rituximab. The other 5 patients were already on optimal doses of tacrolimus and MMF and therefore received two doses of rituximab. Of the six who received rituximab, four (2/3rd) showed a short-term stabilisation of eGFR. The two remaining patients continued to deteriorate at the same rate as before rituximab.

Based on this experience, we made 3 assumptions: a) that 75% of recruits would stabilise within 3 months after optimisation on tacrolimus and MMF; b) that a minority (1/5) who failed to stabilise within 3 months would stabilise within 6 months – this informed the proportion of RCT control arm recruits who would stabilise (i.e. 20%); c) that a majority (4/6) who received Rituximab would show short term stabilisation of eGFR (we rounded up to 70%).Based on these responses, we aimed to recruit 120, to ensure that 30 randomised patients reached the primary EP, which would allow detection of 70% response rate in rituximab-treated vs. 20% response rate in controls, with alpha of 0.05 and beta 0.83. Planned interim per protocol analyses (with stopping rules based primarily on adverse event frequency and secondarily on finding significant differences in response rates), were performed after recruitment of 36 (10 at primary EP) and 61 patients (20 at primary EP). Following the second interim analysis, the DMC halted further recruitment, as the trial was significantly underpowered.

Statistical analysis
Analyses of the primary objective used the per-protocol population as specified in the protocol (provided as a supplement), as recruits who withdrew prior to the primary EP were replaced in the RCT by another eligible participant. Intention to treat analyses were performed as sensitivity analyses where data was available. For continuous outcomes, longitudinal linear mixed models were used. 4 arms of the study (both randomised arms plus 2 observational groups; 'eligible for RCT but not randomised' and 'not eligible for RCT as responded favourably to IS optimisation') were modelled together for efficiency. Recruits not eligible for the RCT on the basis of eGFR<20 were not included in any analyses.
Post-estimation methods were used to extract randomised comparisons between the two arms of the RCT (and for the longitudinal analyses to extract comparisons between the 2 observational groups). These models used all available measures beyond end of phase 1 with a random intercept to account for dependency of repeated measurements, adjusting for end of phase 1 and enrolment values. The randomisation stratifier (binary measure of having deteriorating graft function +/- proteinuria vs. proteinuria only) was also adjusted for. Due to the variation in time measurements collected for each timepoint, time was treated continuously, with observed time of measurements used (months since end of phase 1) rather than the specified discrete timepoints. Post-estimation was used to then estimate differences at these discrete timepoints. These were defined as 4,8,20 and 32 months beyond end of phase 1, to match up with 3-5 months post-randomisation, and 1,2- and 3-years post-enrolment respectively. A treatment x time interaction was included in the model. A quadratic term for time was included in the model to account for non-linearity over time. Models used restricted maximum likelihood (REML) estimation with missing outcomes assumed to be missing at random. A similar approach using generalisations of the longitudinal linear mixed model were planned for non-continuous outcomes where data allowed. Where data was not sufficient, simpler non-parametric models were used, or the data has been described only. No correction was applied to account for multiple testing, either for the co-primary outcomes or for the numerous secondary outcomes or to account for the interim analyses as no corrections were pre-specified. However, all analyses were interpreted conservatively.

Supplementary Results

Per protocol population for the RCT

Control and rituximab groups were randomly matched for enrolment criteria except time from transplantation to enrolment and mean fluorescence intensity (MFI) of donor specific Ab (DSA) (supplementary table 2). All 11 controls reached primary EP so were included in the per-protocol analysis. 3 from the rituximab group were excluded from the per-protocol population (main figure 1). Of the 8 per protocol rituximab recruits who completed phase 2, 7 were administered both doses of rituximab. The second dose was withheld in the eighth after hospitalisation for an unrelated event. The ninth patient, who received both doses, died during phase 2 (see below) but had sufficient data to include in the primary analysis.

Results of the RCT: Primary analysis (second interim timepoint) (supplementary table 4).

Using the co-primary outcomes, there was a small but statistically insignificant estimated mean difference in favour of rituximab in 1/creatinine slopes at 3-5 months post-randomisation from the longitudinal model (0.14 [CI: -0.02, 0.31]; p=0.087). There was no difference in PCR (4.0 [CI: -135.93, 131.49] p=0.95) or odds ratio of formal deterioration of function (0.31 [CI: 0.01-7.13] p=0.46) based on regression analysis of the 1/creatinine plot.

Results of the RCT: Secondary EP analysis.

a) Deterioration in renal function (supplementary table 4).

There were no significant differences in the 1/creatinine slopes for rituximab vs. controls from the longitudinal model, nor in the proportion judged to be deteriorating by formal regression analysis. Four controls and three rituximab-treated patients, all of whom had deteriorating graft function at enrolment, appeared to develop stable or improving graft function. Individual 1/creatinine plots for the per protocol groups, as well as an illustration of changes in ΔeGFR (exploratory) are shown in supplementary figure 3.

b) Change in proteinuria (supplementary table 4).

There were no significant differences in proteinuria when the urine PCR was assessed continuously between groups at any time point. All had a PCR ≥50 at the end of phase 2.

c) Patient survival:

One patient died 15 weeks after the second dose of rituximab and 3 days following emergency replacement of a ruptured aortic valve. Although the event was reported as possibly due to rituximab, at post-mortem only old infective endocarditis was found, with no evidence of new infection.

d) Graft Survival

3/11 control grafts failed, and 2 others withdrew following significant IS reduction in preparation for HD. In the rituximab group, 3/11 grafts failed and another withdrew following IS reduction immediately prior to beginning HD. There was no difference in time to graft failure between the rituximab and control groups using Cox regression [Hazard ratio 1.58, 95% CI 0.33, 7.37]. Log rank tests at each follow up time point also showed no difference by group.

e) Incidence of infections with diagnostic certainty (supplementary table 5).

10 infections (in 5 patients) were documented after rituximab vs. 7 in controls (in 6 patients) (p=NS). As well as endocarditis (see above), one pneumonia with CMV viraemia and one skin abscess were reported as potentially related to rituximab.

f) Incidence of malignancy (supplementary table 5).

There were 2 new neoplasms in the rituximab group vs. 3 in controls (p=NS). In the rituximab group, a stomach adenocarcinoma presented after withdrawal and was reported as potentially related to rituximab.

g) Other adverse events:

See supplementary table 5. Of note, in the RCT population there were 6 adverse drug reactions related to IS optimisation.

h) Changes in circulating DSA (supplementary table 6).
In the RCT population, there were no significant differences in the proportion in either arm with DSA, nor in DSA MFI beyond enrolment. 3 recruits had inadequate tissue typing to identify potential DSA against donor HLA-DQ. 1 in each group developed de novo DSA during phase 3. Three with DSA at enrolment became DSA-negative after optimisation but re-gained DSA in phase 3, 2 of whom received rituximab. There was no association between DSA and graft failure. Individual plots of MFI of DSA for the per protocol groups, as well as an illustration of changes in median MFI for each group (exploratory) are shown in supplementary figure 4.

i)Changes in circulating CD20+ cells (main figure 3).
These are described in the main manuscript

f) Changes in T cell responsiveness to alloantigens by IFNγ ELISPOT.
80 samples from 19 patients were analysed by anti-donor IFNγ ELISPOT. Because of the complexity of the patterns of ELISPOT responsiveness, those from the RCT recruits, and changes related to rituximab, are described as part of the exploratory analysis in the main manuscript.

List of Supplementary Data

Fig. S1. Depletion of subsets in ELISPOT
Fig. S2. Changes in B cells with rituximab – data from whole exploratory group
Fig. S3. Evolution of graft dysfunction for the per protocol control and Rituximab-treated populations.
Fig. S4. Changes in DSA in the per protocol control and Rituximab-treated populations.

Table S1. Recruiting Centres for RituxiCAN-C4 trial.
Table S2. Demographics of the whole study population.
Table S3. Individual patient biopsy features
Table S4. Per protocol analysis of primary and secondary outcomes
Table S5. Adverse events related to rituximab and to optimisation of IS
Table S6. Changes in HLA DSA in patients in the exploratory studyT
Table S7. Dynamic changes in ELISPOT patterns in twenty seven patients with viable and interpretable enrolment samples and at least two viable and interpretable samples at end phase 2 or beyond.
Table S8. Dynamic changes in ELISPOT patterns in the remaining 16 patients from the exploratory analysis.
Supplementary File – The RituxiCAN-C4 study protocol

Supplementary Figure Legends

Figure 1: Depletion of subsets in ELISPOT PBMC

To illustrate the sequential deplation of CD8, CD19 and CD25 cells from PBMC before use in ELISPOT assay. Panels show whole PBMC (A), followed by CD8-depleted PBMC (B: used as the 'baseline' population in ELISPOT, followed by the impact of c0-depleting CD19 (C) , CD25 (D) and both CD19 and CD25 (E). Importantly, when CD25 cells are depleted, CD25++ cells are completely depleted but a significant number of CD25+ cells remain (bottom two rows).

Figure 2: Changes in B cells with rituximab – data from whole exploratory group

Graphs are box and whisker plots showing median with interquartile range (IQR) with whiskers showing upper and lower limits and outliers indicated as single data points. Means are represented with 'x'. White; Control group exploratory study, Grey; per protocol rituximab group. P values by Mann Whitney U test. Time points: 0= enrolment sample. EP-1; End phase 1. 0-36=months post enrolment. Rituximab administered between EP-1 and 12 months. 'N' refers to the number of samples at each time point.

A-F: Changes in B cells

A: Absolute numbers of B cells per uL of serum.

B-F: Flow cytometric analysis of the proportion of B cell subpopulations against time.

B: CD27-negative B cells as proportion of total CD19+ cells

C: CD27+ B cells as proportion of CD19+ cells

D: CD38loCD24lo cells as proportion of CD27- cells (naïve B cells).

E: CD38++CD24++cells as proportion of CD27- cells (Transitional T1 cells).

Median absolute number of T1 per μL is shown beneath each column.

F CD38+CD324+ cells as proportion of CD27- cells (Transitional T2 cells).

Figure 3: Evolution of graft dysfunction for the per protocol control and Rituximab-treated populations.

A&B: Graphs show 1/creatinine plots against time (days) for the people included in the primary endpoint analysis of the RCT in control (A) or Rituximab-treated (B) groups. All creatinine data included. Time 0 is the day of enrolment. Dots are colour coded to indicate the phase of the study. Green =pre-enrolment. Yellow = phase 1. Red = Phase 2. Blue = phase 3. Graphs annotated with time of Rituximab administration and day of withdrawal or graft failure. A negative slope on this graphical representation indicates deteriorating graft function.

C: From the exploratory analysis. Box and whisker plot showing median ΔeGFR (normalised to enrolment eGFR of 0), with interquartile range (IQR) with whiskers showing upper and lower limits and outliers indicated as single data points. Means are represented with 'x'. White; control group, Grey; Rituximab group. P values by Mann Whitney U test

Time points: 0= enrolment sample. EP-1; End phase 1. EP-2; End phase 2. 0-36=months post enrolment

Figure 4: Changes in DSA in the per protocol control and Rituximab-treated populations.

A: Box and whisker plots showing median cumulative MFI of all DSA with interquartile range (IQR) with whiskers showing upper and lower limits and outliers indicated as single data points. Means are represented with 'x'. White; control group, Grey; Rituximab group. Time points: 0= enrolment sample. EP-1; End phase 1. EP-2; End phase 2. 9-36=months post enrolment All comparisons p=NS (Mann Whitney U test)

B&C: Individual plots from each patient in control (B) or Rituximab-treated (C) groups.

Time points: enrol= enrolment sample. EP-1; End phase 1. EP-2; End phase 2. V1-V10 correspond to 9-36 months in A.

Supplementary Table 1 – Recruiting Centres for RituxiCAN-C4 trial.

Centre	Recruited patients
West London Renal and Transplant Centre, London	17
Guy’s & St Thomas’ NHS Foundation Trust, London	30
St Jame's University Hospital, Leeds	2
University Hospital, Birmingham	4
University Hospital of Wales, Cardiff	6
Epsom and St Helier University Hospitals Trust	1
NHS Greater Glasgow and Clyde Trust Renal Unit, Glasgow	2
Royal Free Hospital, London	0
Hull Royal Infirmary, Hull	0
Addenbrookes University Hospital, Cambridge	0
Kent and Canterbury Hospital, Canterbury	0
Royal Preston Hospital, Preston	0
King’s College Hospital, London	0

Supplementary table 2 – Baseline characteristics of the fifty-nine patients who passed eligibility criteria to enter Phase 1.

	Included in Exploratory Analysis					Not included in exploratory analysis		
	Poor response to IS[1]			Good response to IS (N=15)	P[2]			
	RCT		Not randomised (N=9)					
Baseline Characteristic	Control (N=11)	Rituximab[3] (N=12)				[4]eGFR <20 (N=5)	P[5]	Withdrawn Phase 1 (N=7)
Age (Years - Median (IQR))	50 (15.5)	44 (24.5)	40 (7)	46 (13)	*	34 (13)	*	59 (18)
Male n (%)	8 (73)	8 (67)	7 (77)	9 (60)	*	3 (60)	*	5 (71)
Ethnicity n (%)								
Asian	1 (9)	2 (17)	1 (11.1)	2 (13.3)	*	2 (40)	*	1 (14.3)
Black	-	1 (8)	2 (22.2)	2 (13.3)	*	-	*	1 (14.3)
White	10 (91)	9 (75)	6 (66.6)	11 (74.3)	*	3 (60)	*	5 (71.4)
Cause of renal failure								
DM	-	-	-	-	*	-	*	1
APKD	2	-	-	-	*	-	*	1
GN	4	3	3	7	*	1	*	1
SLE	-	1	-	1	*	-	*	-
HT	-	1	1	1	*	-	*	2
Congenital[6]	2	4	2	1	*	-	*	-
TIN[7]	1	1	3	1	*	3	*	-
Cystinosis	1	1	-	-	*	-	*	-
HUS	-	-	-	1	*	-	*	-
CNI toxicity	-	-	-	1[8]	*	-	*	1[9]
Unknown / not recorded	1	1	-	2	*	1	*	1

[1] All who were eligible for RCT + the patient (G008) who developed a contraindication to Rituximab during phase 1
[2] P value, comparing good response to optimised IS (N=15) to all poor response to optimised IS, eGFR>20 (N=32)
[3] Includes 3 patients who did not receive Rituximab
[4] eGFR <20 at end of phase 1, therefore not eligible for RCT
[5] P value, comparing poor response to optimised IS, eGFR>20 (N=32) to poor response to optimised IS, eGFR <20 (n=5)
[6] Including Alports
[7] Including chronic pyelonephritis
[8] Heart transplant recipient
[9] Liver transplant recipient

Transplant history n ;								
Deceased	9	8	5	6	*	2	*	5
LRD	2	3	3	6	*	2	*	1
LURD	-	1	1	3	*	1	*	1
Previous transplants								
0	9	10	7	14	*	4	*	6
1	2	2	2	1	*	1	*	1
Time from Tx (years-median (IQR))	19.6 (7.2)	5.6 (6.8)	14.8 (10)	16.6 (12.7)	*	11.6 (6.9)	*	6.8 (2.5)
HLA MM (Mean (SD))								
Overall	3.3 (1.3)	2.3 (1.6)	3 (1.3)	3.3 (1)	*	2 (0.8)		2.4 (0.8)
A	1.3 (0.8)	0.9 (0.5)	1.2 (0.4)	1 (0.8)	*	0.5 (0.6)	*	1 (0.5)
B	1.3 (0.6)	0.9 (0.7)	1.1 (0.8)	1.3 (0.7)	*	0.75 (0.5)	*	0.6 (0.5)
DR	1 (0.5)	0.5 (0.5)	0.7 (0.5)	1.2 (0.5)	ø	0.75 (0.5)	*	0.9 (0.7)
							*	
HLA Ab status								
CRF (Mean (SD))	49.4	42.5 (41.1)	54.9 (39.3)[11]	42.7 (37.7)	*	57.8 (34)[11]	*	47 (55)[11]
DSA+ n(%)	(37.9)	6 (50)	4 (50)	11 (74.3)	*	4 (100)	*	3 (43)
-Class I	7 (64)	3 (25)	3 (33.3)	3 (20)	*	3 (75)	*	2 (29)
-Class II	4 (36)	1 (8.3)	0	5 (33.3)	*	1 (25)	*	0
-Both	2 (18)	2 (16.7)	1 (11.1)	3 (20)	*	0	*	1 (14)
-NA	1 (9)	6 (50)	4 (44.4)	4 (26.7)	*	0	*	0
DSA MFI[10]	5 (45)	6822	4317 (7952)	6758 (8998)	*	6561	*	15793 (17946)
(Mean (SD))	1923	(7761)				(5263)		
	(2724)							
Enrolment biopsy scores – median (IQR)								
C4d glom[12]	3 (1)	2 (1.3)	3 (1)	3 (1)	*	2 (1)	*	3 (0.5)
Banff C4d (PTC)	3 (1)	2 (3)	2 (0)	2 (2)	*	2 (3)	*	2 (1.5)
Bannf g	2 (1.5)	2 (1.3)	2 (2)	1(2)	*	1 (1)	*	1 (1)
Banff ptc	2 (2)	1 (1.3)	0 (2)	1 (1)	*	2 (0)	*	1 (1)
Banff cg	1 (1.5)	2.5 (2)	2 (2)	1 (1)	*	2 (2)	*	3 (1)

[10] Cumulative - includes those with DSA=0
[11] No HLA Ab data available on 3 recruits
[12] Scored as C4d PTC

Banff cv	2 (1)	1 (2)	1 (1)	1.5 (1)	*	1 (2)	*	3 (1.5)
Banff ct	2 (1)	1 (1)	1 (1)	1 (1)	*	1 (1)	*	1 (0.5)
Banff ci	2 (1)	1 (1)	1 (1)	1 (1)	*	1 (1)	*	1 (0.5)
TA/IF (%)	25 (7.5)	25 (21)	25 (15)	20 (20)	*	25 (10)	*	30 (17.5)
Baseline immunosuppression n (%)								
CNI	8 (73)	11 (92)	6 (67)	15 (100)	*	5 (100)	*	7 (100)
Tac	4 (37)	10 (83)	5 (56)	8 (53)	*	3 (60)	*	5 (71)
CsA	4 (37)	1 (8)	1 (11)	7 (47)	*	2(40)	*	2 (29)
Anti-metabolite	8 (73)	11 (92)	8 (89)	14 (93)	*	5 (100)	*	6 (86)
MPA	5 (45)	10 (83)	7 (78)	9 (60)	*	4 (80)	*	6 (86)
Azathioprine	3 (27)	1 (8)	1 (11)	5 (33)	*	1 (20)	*	0
Baseline renal function (Mean (SD))								
Creatinine[13]	183 (36)	182 (48)	189.7 (74)	168.7 (44.8)	*	260.8	†	236.3 (37.4)
eGFR[14]	34.9 (8.6)	37.3 (8)	41.7 (17.7)	38.6 (11.3)	*	(32.8)	†	25 (3.6)
1/creat slope	-0.21 (0.3)	-0.17 (0.2)	-0.08 (0.13)	-0.07 (0.07)	*	22.8 (1.6)	*	-0.146 (0.08)
Formally deteriorating	9 (82)	10 (83)	4 (44)	10 (67)	*	-0.27 (0.32)	*	7 (100)
PCR[15] (Mean (SD))	201 (206)	242 (168)	188 (280)	74 (74)	†	5 (100)	*	305 (245)
PCR >50	8 (73)	11 (92)	8 (89)	8 (53)	ø	237 (138)	*	5 (71)
						5 (100)		
Post-optimisation (pre-randomisation) immunosuppression								
Tac n (%)	10 (91)	12 (100)	9 (100)	15 (100)	*	5 (100)	*	-
MPA n (%)	9 (82)	12 (100)	9 (100)	15 (100)	*	5 (100)	*	-
Tac level (Mean (SD))	5.9 (3.4)	5.6 (2.3)	4.4 (2.4)	7.0 (2.2)	ø	4.8 (2)	*	-
End Phase 2 medication[16]								
Tac n (%)	10 (91)	8 (100)[19]						
Tac level Mean (SD)	5.9 (3.5)	5 (3.2)						

[13] µmol/L
[14] mls/min/1.73 m^2
[15] mg/mmol
[16] In those RCT patients reaching primary EP.

MPA n (%)	9 (82)	8 (100)						
On ACE-I n (%)	6 (55)	3 (38)						
On ARB n (%)	8 (73)	6 (75)						

*P=NS
†P≤0.005
øP<0.05
1º renal diagnosis: DM=diabetes mellitus: APKD=adult polycystic kidney disease: GN=glomerulonephritis: SLE=systemic lupus erythematosus: HT=hypertension: TIN=tubulointerstitial nephritis: HUS=haemolytic uraemic syndrome: CNI=calcineurin inhibitor. *Tx type:* LURD: living unrelated donor. LRD: living-related donor. *HLA MM*; Number of Class I (A,B) and Class II (DR) mismatches. Two patients (B003 and W007) had their transplants abroad and tissue typing was unavailable. *HLA Ab status:* CRF=calculated reaction frequency. DSA=donor specific antibody. MFI=cumulative median fluorescence intensity. Means are shown for whole group, including those with MFI of 0. *Enrolment biopsy scores:* g=glomerular inflammation. ptc=peritubular capillary inflammation. c= chronic scores. TA/IF=tubular atrophy/interstital fibrosis. *Immunosuppression*; Tac = Tacrolimus; CsA= ciclosporin; MPA= Mycophenolic acid or mycophenolate mofetil. *Baseline renal function:* eGFR= estimated glomerular filtration rate (4 value MDRD). Formally deteriorating =meet criteria for deteriorating function based on analysis of 1/creatinine plot. *Medication*: ACE-I= angiotensin converting enzyme inhibitor: ARB= angiotensin II receptor blocker.

Supplementary table 3 – individual patient biopsy features

ID	C4d g§	C4d ptc	g	ptc	cg	cv	ct	ci	TA/IF%	other features
Withdrawn phase 1										
W001*Ω	2	2	2	1	3	3	1	1	20	TG on EM
W005* Ω	1	2	1	0	1	3	1	1	15	
G004ø Ω	3	3	1	2	3	2	2	2	40	
G005ø Ω	3	3	2	3	2	3	3	3	70	
G020º Ω	3	1	2	1	3	3	1	1	30	
C006* Ω	3	1	1	2	2	1	1	1	<25	
X002† Ω	3	0	1	1	3	1	1	1	10	
Not eligible for RCT – good response to optimised IS										
W002†Ω	0	3	0	2	0	1	2	2	30	Chronic TMA
W003†¥	2	0	1	0	1	2	2	2	30	
W004ø Ω	2	1	0	0	0	2	1	1	15	PTCBMML on EM
W009ø Ω	3	2	2	1	1	1	1	1	10	
W012ø Ω	2	2	0	1	0	2	1	1	10	PTCBMML on EM
W013ø¥	3	0	0	1	0	0	3	3	50	PTCBMML on EM
W014ø Ω	3	2	1	0	2	2	1	1	10	PTCBMML on EM
W016ø Ω	2	2	2	2	3	1	1	1	15	
G014† Ω	3	2	2	1	1	3	2	2	25	
G018ø Ω	3	1	1	1	1	2	1	1	10	
G019øΩ	3	3	1	2	1	1	0	1	10	
G022øΩ	3	3	2	2	1	2	2	2	30	
G024øΩ	0	0	2	2	3	1	1	1	20	
G030†Ω	0	0	0	3	3	-	3	3	80	No medium/larger calibre arteries to score
C001øΩ	3	3	1	1	1	1	1	1	<25	
Not eligible for RCT – GFR<20 or contraindication to rituximab										
G001øΩ	2	2	1	2	2	3	2	2	40	
G006øΩ	2	3	0	2	1	2	1	1	20	

G008øΩ	3	3	2	3	3	-	2	2	30	TG on EM. No medium/larger calibre arteries to score
G028ø¥	0	0	2	3	0	0	1	1	25	
B003*Ω	3	3	1	2	3	1	3	3	30	
B004ø¥	3	0	0	0	3	0	1	1	15	
Eligible for RCT – not randomised										
W011øΩ	1	2	0	0	1	1	3	3	50	
W015†Ω	2	2	2	0	3	1	1	1	10	PTCBMML on EM
W017†Ω	2	2	3	0	2	1	1	1	15	
B001øΩ	3	2	0	0	1	1	0	0	5	TG on EM
G015øΩ	3	0	2	3	3	2	1	1	25	PTCBMML on EM
G016†Ω	0	2	0	0	1	2	3	3	50	IgA deposition, no mesangial proliferation
C002†Ω	3	3	2	1	3	0	1	1	<25	
C005øΩ	3	2	1	2	2	2	1	1	<25	
Eligible for RCT - randomised to control										
W006†Ω	3	1	2	1	2	1	1	1	20	
W008†Ω	2	0	2	0	1	2	2	2	25	
G002øΩ	3	3	0	0	3	2	2	2	30	Chronic TMA
G007øΩ	3	3	1	2	1	0	1	1	15	
G011ø¥	3	3	0	2	0	2	2	2	30	
L001†Ω	1	1	0	0	1	1	3	3	50	PTCBMML on EM
G013†Ω	3	3	2	2	2	2	1	2	25	t2
G027øΩ	0	0	2	0	3	0	1	1	10	TG and PTCBMML on EM
X001øΩ	3	3	2	2	3	3	2	2	30	
G023øΩ	2	2	2	2	1	3	2	1	30	
C003øΩ	3	3	1	2	1	1	1	1	<25	
Eligible for RCT - randomised to Rituximab										
W010øΩ	2	2	3	0	3	1	1	1	15	
G003øΩ	3	3	3	1	3	2	1	1	20	
G009†Ω	3	3	3	3	3	-	1	1	15	No medium/larger calibre arteries to score
G010øΩ	3	3	2	3	3	-	2	2	40	No medium/larger calibre arteries to score
G012øΩ	3	0	3	2	2	3	3	3	60	
G017†Ω	2	2	3	1	3	1	2	2	40	

B002†¥	3	0	0	0	1	0	1	1	25	IgA deposition, no mesangial proliferation
L002ø¥	3	3	1	1	0	0	1	1	25	
G025øΩ	3	0	2	2	3	3	2	2	50	t3 i3 v0
W007†¥	1	2	1	1	0	2	2	2	40	
G029†Ω	0	0	2	3	1	0	1	1	25	
C004†Ω	1	1	2	1	1	0	0	0	0	

§ Glomerular C4 scored as for PTC. Where score is 2, C4d staining affected 25-50% of capillaries
Enrolment DSA status: *not known; † negative; Ø positive
BANFF 13: Ω meet histological criteria ¥ do not meet histological criteria.
TG: transplant glomerulopthy; EM: electron microscopy; PTCBMML: peritubular capillary basement membrane multilayering; TMA: thrombotic microangiopathy.

Supplementary table 4: Per protocol analysis for primary outcomes at primary and secondary EPs

**Outcome/ Timepoint***	**3 - 5 months post-randomisation**	**12 months post-enrolment**	**24 months post-enrolment**	**32 months post-enrolment**
	Estimated mean difference: Rituximab versus Control (95% CI)			
**1/Creatinine slope[1]**	0.14 (-0.02, 0.31) SE=0.08 p=0.087	0.14 (-0.02, 0.30) SE=0.08 p=0.08	0.13 (-0.03, 0.28) SE=0.08 p=0.111	0.11 (-0.08, 0.30) SE=0.1 p=0.244
**Protein: Creatinine Ratio (PCR)[2]**	4.00 (-135.93, 131.49) SE=69.59 p=0.954	24.15 (-111.21, 162.00) SE=71.93 p=0.737	84.60 (-57.73, 256.79) SE=117 p=0.472	145.05 (-37.70, 390.15) SE=184 p=0.431
	Estimated odds ratio: Rituximab versus Control (95% CI, p-value)			
**Formal binary criteria for deterioration[3]**	0.31 (0.01, 7.13) SE=0.5 p=0.464	0.33 (0.02, 5.97) SE=0.49 p=0.456	0.42 (0.01, 13.48) SE=0.75 p=0.626	0.53 (0.001, 107.38) SE=1.44 p=0.816

*Estimates extracted from models at 4,8,20 and 32 months post-baseline respectively to approximately match pre-specified timepoints.

[1]Estimated using a longitudinal linear mixed-effects model, with 4 arms of the study modelled together (including the 2 observational non-randomised arms) and randomised comparisons extracted using post-estimation methods. All available post-baseline (time 2, end of phase 1) observations included with a random intercept to account for dependency of repeated measurements, adjusting for baseline (time 2) and enrolment (time 1) values and randomisation stratifier. Time treated continuously with observed time of measurements used. A treatment x time interaction was included in the model. A quadratic term for time was included in the model to account for non-linearity over time. Model estimated using restricted maximum likelihood (REML) estimation, with missing outcomes assumed to be missing at random.

[2]Estimated the same way as for the 1/Creatinine slope outcome but with bootstrapped bias-corrected CIs (1000 repetitions) reported as residuals were non-normal.

[3]Estimated the same way as for the 1/Creatinine slope outcome but using generalisation to binary data (mixed-effects logistic regression) to estimate odds ratios, not including the randomisation stratifier and time 1 and time 2 observations as covariates for convergence and with robust option for standard errors.

Supplementary table 5 – Adverse events organised either according to events seen in RCT (top rows), or by events seen after optimisation of IS (bottom rows).

Group	Systemic Infections	Cardio-vascular	Respir-atory	Gastro-intestinal	Genito-urinary	Endocrine /Metabolic	Haemato-logical	Musculo-skeletal	Neuro-logical	Dermato-logical	Allergic	Ear Nose Throat	IMP-related	Total
**RCT-Control**														
Neoplastic	-	-	-	-	-	-	-	-	-	3	-	-	-	3
Infective – suspected<br>Infective – no diagnostic doubt	1<br>1	-	3	2 (2)	1<br>3	-	-	-	-	-<br>3	-	-	-	7(2)<br>7
Non-IMP drug-related	-	1	-	1	-	-	-	-	2	-	-	-	-	4
Other	-	2	1	5 (2)	1 (1)	2 (2)	6 (1)	1	-	1	-	-	-	19 (6)
**Total**	**3**	**3**	**4**	**8 (4)**	**5 (1)**	**2 (2)**	**6 (1)**	**1**	**2**	**6**	**0**	**0**	**0**	**40 (8)**
**RCT-Rituximab**														
Neoplastic	-	-	-	1 (1)	-	-	-	-	-	1	-	-	1 (1)	2 (1)*
Infective – suspected<br>Infective – no diagnostic doubt	1	-	5¶ (2)<br>2# (2)	-	-<br>2§ (1)	-	-	-<br>1	-	1<br>5 (1)	-	-	-<br>3 (2)	7<br>10 (4)*
Non-IMP drug-related	-	-	-	-	-	1	-	-	-	1†	-	-	-	2
Other	-	1	-	6¶ (1)	-	4 (1)	4 (1)	4	4 (1)	2	-	-		25 (4)
**Total**	**1**	**1**	**7 (2)**	**7 (2)**	**2 (1)**	**5 (1)**	**4 (1)**	**5**	**4 (1)**	**10 (1)**	**0**	**0**	**4**	**46 (9)***
**Good response to optimised IS (PCR<50, stable creatinine)**														
Neoplastic	-	-	-	-	-	-	-	-	-	-	-	-	-	0
Infective	2 (2)	-	8	1	6 (1)	-	-	-	-	15 (3)	-	-	-	32
Non-IMP drug-related	-	-	-	4 (1)	-	1	-	-	-	1 (1)	-	-	-	6
Other	-	8 (3)	1 (1)	7 (2)	4 (1)	5	4	1	1	3 (1)	-	2	-	36
**Total**	**2**	**8 (3)**	**9 (1)**	**12 (3)**	**10 (2)**	**6**	**4**	**1**	**1**	**19 (5)**	**0**	**2**	**0**	**74**
**Poor response to optimised IS (PCR>50 ± deteriorating creatinine, eGFR>20)¥**														
Neoplastic	-	-	-	1 (1)	1	-	-	-	-	4	-	-	1 (1)	6 (1)*
Infective	4	-	14 (2)	2 (2)	6 (1)	-	-	1	-	10 (2)	-	-	3 (2)	37 (6)*
Non-IMP drug-related	-	1	-	1	-	1	1	-	2	2	1	-	-	9
Other	-	8 (4)	1	15 (5)	1 (1)	8 (3)	10 (2)	7	4 (1)	5	-	-	-	59 (16)
**Total**	**4**	**9 (4)**	**15 (2)**	**19 (8)**	**8 (2)**	**9 (3)**	**11 (2)**	**8**	**6 (1)**	**21 (2)**	**1**	**0**	**4 (3)**	**111 (23*)**
**P values**	NS	**NS**	NS	NS	NS	**NS**	NS	**NS**	NS	**NS**	**NS**	**NS**	**NS**	**NS**

AEs meeting criteria for SAE are in (parentheses)
*The adverse events classified as Investigational Medical Product (IMP)-related are only counted once in the total.
¶ includes a chest infection and non-drug-related diarrhoea in a patient who withdrew in phase 2 before receiving Rituximab.
# includes severe pulmonary oedema due to acute aortic valve rupture in a patient, which contributed to patient death.
§includes a UTI in a patient who withdrew during phase 2 post-rituximab
† includes angioedema secondary to Ramipril in a patient who did not receive rituximab
¥ this group includes the patients in the control and rituximab-treated groups

Supplementary Table 6: Changes in Donor specific antibody (DSA) Median Fluorescence intensity (MFI) in patients included in the exploratory study

Recruit number	Specificities of DSA (DSA with MFI<2000)	Enrol	End Phase 1	End Phase 2	Phase 3									
						End Year 1				End Year 2				End Year 3
Good response to optimisation of IS														
W002	None*	0	-		0	0	0	0	0	0	0	-	-	-
W003	CW4	0	1814		0	881	0	0	2377	1758	0	0	0	0
W004	B44, DR53 (Cw2)	7280	15587		11214	16552	17270	14228	11724	10683	16055	12984	18644	16791
W009	B44, DR53, DQ8 (B60, Cw5)	6271	3311		3049	14124	12343	0	13103	11486	0	0	10870	-
W012	DQ3	15000	10000		17330	10872	16088	17642	13903	16350	-	-	8763	-
W013	(A3)	400	0		0	-	0	0	-	-	-	-	-	
W014	DQ4 (B60)	14805	12133		12710	15772	10932	15160	11220	14179	-	9756	15010	-
W016	(B35, DR1)	750	695		981	-	-	-	-	-	-	-	-	-
G014	A2 (A1)	0	-		0	0	0	0	0	4214	3450	4052	0	0
G018	DQ7	9655	0		7241	8255	9627	5830	8532	6546	5322	6260	8642	8529
G019	B44, Cw5	6526	5833		7428	3969	6440	5886	2468	7331	2010	-	2915	1893
G022	DQ6	4898	0		0	0	0	-	0	-	4277	0	-	-
G024	A28, A11, B14, DQ3	33494	46868		49299	32692	46397	33556	-	-	-	-	-	-
G030	DQ3	0	9867		12090	0	-	-	-	-	-	-	-	-
C001	DR13, DR52 (A3, B61)	3200	10132		-	-	-	-	-	-	-	-	-	-
Poor response to optimisation of IS														
W006 (RCT-C)	(B37)	0	0	0	0	0	0	0	0	0	0	0	1908	-
W008 (RCT-C)	None	0	0	0	0	0	0	0	0	0	0	0	0	-
G002 (RCT-C)	A1, (Cw7)	2784	2199	-	0	2837	-	-	-	-	-	-	-	-
G007 (RCT-C)	A2, DR12, (Cw7)	8845	15450	13682	-	10126	11413	16469	20864	14495	-	-	-	-
G011 (RCT-C)	DR17*	1601	8568	-	-	-	-	-	-	-	-	-	-	-
L001 (RCT-C)	None*	0	0	-	-	-	-	-	-	-	-	-	-	-
G013 (RCT-C)	None*	0	0	0	0	0	0	0	0	0	0	0	0	0
G027 (RCT-C)	(B8)*	750	1020	-	750	-	800	-	-	-	-	-	-	-
X001 (RCT-C)	B7	8714	14850	16049	-	12658	-	-	16140	-	-	-	-	-
G023 (RCT-C)	DR53*	3591	4426	4500	-	-	-	-	-	-	-	-	-	-

C003 (RCT-C)	A2	3592	-	-	-	-	-	-	-	-	-	-	-	-
Given Rituximab														
W010 (RCT-R)	A2, DR53	6617	0	0	8019	0	0	-	-	-	-	-	-	-
G003 (RCT-R)	B8	15246	10358	13934	13335	-	9486	-	12518	9076	10996	12846	10384	8979
G009 (RCT-R)	None*	0	0	-	-	-	-	-	-	-	-	-	-	-
G010 (RCT-R)	A1 (B8)	14010	6632	-	-	-	-	-	-	-	-	-	-	-
G012 (RCT-R)	A2, A23, DQ2 (B44)	20711	12278	16573	15570	10999	8277	14028	16279	12566	-	-	-	-
G017 (RCT-R)	Cw4	0	0	2693	2542	1739	0	1200	-	-	-	-	-	-
B002 (RCT-R)	None	0	0	-	0	-	0	0	-	0	-	0	-	-
L002 (RCT-R)	A1	12460	10717	-	-	8697	10010	12586	10380	9417	-	-	-	-
G025 (RCT-R)	DR53, DQ7	12824	0	4308	14265	-	16972	15402	6113	-	-	-	-	-
W007 (RCT-R†)	None	0	0	-	-	-	-	-	-	-	-	-	-	-
G029 (RCT-R†)	DR53	0	1656	-	0	2147	-	-	-	-	-	-	-	-
C004 (RCT-R†)	None	0	-	-	-	-	-	-	-	-	-	-	-	-
G008 (NR)	A11, Cw12 (A32, B18, B52, DR7)	22867	24630		20992	-	-	20655	-	-	-	-	-	-
W011 (NR)	A2	3912	9590		12084	11096	11612	15616	19752	13951	13066	-	12255	5055
W015 (NR)	DQ2	0	4067		3813	-	-	-	-	-	-	-	-	-
W017 (NR)	B7 B60	0	0		0	0	10481	0	0	0	0	0	-	-
B001 (NR)	A1 (DQ8)	450	4240		3801	-	6168	-	17856	-	-	4904	12435	14193
G015 (NR)	DR14 (Cw2, DR52)	-	4192		3185	2667	2315	3254	3039	6414	9100	3749	7396	-
G016 (NR)	(DQ6)	0	1603		1220	0	0	1993	1855	0	0	400	-	-
C002 (NR)	none	0	-		-	-	-	-	-	-	-	-	-	-
C005 (NR)	B40	7307	-		-	-	-	-	-	-	-	-	-	-

Specificities in (parentheses) = MFI <2000:
RCT-C – randomised to control group
RCT-R – randomised to rituximab group: † Not given rituximab
NR – not randomised
G002 – Cw7 only at enrol and end year 1. G007 - all separate DSA intermittently positive throughout. G010 - B8 present at enrolment only. G012 – DQ2 only at enrol, B44 only during phase 3 year 2.
W004 – DR53 at all time points, B44 detected at EP-1 and T6
W009 – DR 53 at enrol, EP-1, T1. DQ8 at T2, T3, T5, T6 and T9. B44 only at T9. T8 No DSA.
* No donor DQ typing available. Linkage disequilibrium suggests there may be potential DSA to donor DQ.

Supplementary Table 7 – Dynamic changes in ELISPOT patterns, in 27 patients in the exploratory study who had viable and interpretable enrolment samples, with ≥2 at or beyond end of phase 2. (Refer to main table 4 and figure 5D for how these patterns relate to trends in eGFR)

	Enrolment	End Phase 1	9/12 (or End phase 2)	End year 1	End Year 2	End year 3
ID	Anti-donor response	Anti-donor response	Anti-donor response	Anti-donor response	Anti-donor response	Anti-donor response
	No Non-regulated B-dependent anti-donor IFNγ at or beyond phase 2					
W003 (GRO)	No response	No response	No sample	No response	No response	No response
W012 (GRO)	No response	CD19+ Regulated B-dependent*	No sample	No response	Regulated B-dependent†	No response
B001 (NR)	No response	No response	No sample	No response	No sample	No response
W014 (GRO)	No response	No response	No sample	No response	No response	No response
G014 (GRO)	No response	No response	No sample	No response	No response	No response
W002* (GRO)	Unreg B-dependent	No response	No sample	CD25+ Regulated B-dependent	No response	Graft failure
W009 (GRO)	No response	No sample	No sample	No response	No response	Regulated B-dependent†
W011 (NR)	No response	No response	No sample	CD19+ B-regulated	Regulated B-dependent†	CD19+ B-regulated
G018 (GRO)	No response	No response	No sample	No response	No response	No response
X001 (RCT-C)	No response	No response	No response	No response	No response	No sample
G007 (RCT-C)	No response	No response	No response	No response	No response	No sample
G024 (GRO)	No response	No response	No sample	CD25+ CD19+ B-regulated*	No response	No sample
G015 (NR)	No response	No response	No sample	No response	CD25+ Regulated B-dependent	Withdrawn
W006 (RCT-C)	Unreg B-dependent	CD19+ Regulated B-dependent*	CD19+ B-regulated	No response	No response	No response
G013 (RCT-C)	CD25+ Regulated B-dependent	No response	No response	CD25+ CD19+ B-regulated*	No response	No response
W008 (RCT-C)	CD25+ Regulated B-dependent	No response	CD25+ CD19+ B-regulated*	No response	No response	CD25+ CD19+ B-regulated*
G022* (GRO)	Unreg B-dependent	No response	No sample	CD19+ B-regulated	No response	CD25+ Regulated B dependent
L002 (RCT-R)	No response	CD25+ Regulated B-dependent	No response	No sample	No response	Graft Failure
G012 (RCT-R)	CD25+ Regulated B-dependent	No response	No response	No response	No response	Withdrawn

G003 (RCT-R)	CD19+ B-regulated	CD25+ Regulated B-dependent	No response	No response	No response	No response
B002 (RCT-R)	Regulated B-dependent†	Regulated B-dependent†	No response	No sample	CD25+ Regulated B-dependent	No response
	At least 1 sample showing non-regulated B-dependent anti-donor IFNγ at or beyond phase 2					
G016 (NR)	Unreg B-dependent	No response	No sample	Unreg B-dependent	CD25+ CD19+ B-regulated*	CD25+ Regulated B-dependent
W017 (NR)	No response	Regulated B-dependent†	No sample	CD19+ B-regulated	Unreg B-dependent	Graft failure
G002 (RCT-C)	Unreg B-dependent	CD25+ CD19+ B-regulated*	No response	Unreg B-dependent	Graft Failure	
W010 (RCT-R)	Unreg B-dependent	CD19+ B-regulated	Regulated B-dependent†	Unreg B-dependent	Graft Failure	
G025 (RCT-R)	Unreg B-dependent	No response	Unreg B-dependent	No response	No response	No response
G017 (RCT-R)	CD25+ Regulated B-dependent	Unreg B-dependent	No response	No sample	Unreg B-dependent	No sample

W002* and G022* - given Rituximab outwith the trial protocol in year 2.
GRO – good response to optimised IS
RCT-C – randomised to control group
RCT-R – randomised to rituximab group
NR – not randomised
CD25+ regulated B-dependent – Evidence of B-dependent anti-donor responses only when CD25+ cells depleted (shaded blue)
CD19+ Regulated B-dependent*– Evidence of Bdep when CD25+ present, but Breg when CD25+ absent (shaded blue)
Regulated B-dependent*† – Evidence of CD25+ or CD19+ regulation at one antigen dose, but B-dependent responses at a second antigen dose (shaded blue).
CD19+ B-regulated – Evidence of anti-donor reactivity when CD19+ cells depleted, when CD25+ cells present (shaded green)
CD25+ CD19+ B regulated* – Evidence of Breg when CD25+ absent, Suppression of all anti-donor reactivity when CD25+ present (shaded green)
Unregulated B-dependent – Anti-donor activity reduces (≥20%) when B cells depleted. No evidence of regulation (shaded orange)

Supplementary table 8: Dynamic ELISPOTS from the 16 remaining patients in the exploratory study.

	Enrolment	End Phase 1	9/12 (or End phase 2)	End year 1	End Year 2	End year 3
ID	Anti-donor response	Anti-donor response	Anti-donor response	Anti-donor response	Anti-donor response	Anti-donor response
L001 (RCT-C)	CD19+ B-regulated	No response	No sample	No sample	Graft failure	
G027 (RCT-C)	No response	No response	No sample	No response	No sample	Withdrawn
G023 (RCT-C)	Unreg B-dependent	CD25+ Regulated B-dependent	No response	Withdrawn		
C003 (RCT-C)	No response	No samples	No sample	No sample	No sample	No sample
G009 (RCT-R)	No response	No response	Graft failure			
G010 (RCT-R)	No response	No response	No response	Graft failure		
W004 (GRO)	Non-viable	Non-viable	No sample	Reactive – not interpretable further	No response	No response
W013 (GRO)	No response	Unreg B-dependent	No sample	No response	Withdrawn	
W016 (GRO)	No response	No response	No sample	Withdrawn		
G030 (GRO)	No response	No response	No sample	No response	No sample	Graft failure
C001 (GRO)	No response	No response	No sample	No response	No sample	Graft failure
G008 (NR)	No response	No response	No sample	No response	Graft Failure	
W015 (NR)	No response	No response	No sample	Withdrawn		
C002 (NR)	CD19+ B-regulated	No sample	No sample	No sample	No sample	No sample
C005 (NR)	No response	No sample	No sample	No sample	No sample	No sample
G029 (RCT-R†)	No response	No response	No sample	CD19+ B-regulated	Withdrawn	

These samples are included in the analyses contained in main table 2 and 3, but not in the analysis included in main table 4 and figure 5D.
GRO – good response to optimised IS
RCT-C – randomised to control group
RCT-R – randomised to rituximab group: † Not given rituximab
NR – not randomised
CD25+ regulated B-dependent – Evidence of B-dependent anti-donor responses only when CD25+ cells depleted (shaded blue)
CD19+ Regulated B-dependent*– Evidence of Bdep when CD25+ present, but Breg when CD25+ absent (shaded blue)
Regulated B-dependent*† – Evidence of CD25+ or CD19+ regulation at one antigen dose, but B-dependent responses at a second antigen dose (shaded blue).
CD19+ B-regulated – Evidence of anti-donor reactivity when CD19+ cells depleted, when CD25+ cells present (shaded green)
CD25+ CD19+ B regulated* – Evidence of Breg when CD25+ absent, Suppression of all anti-donor reactivity when CD25+ present (shaded green)
Unregulated B-dependent – Anti-donor activity reduces (≥20%) when B cells depleted. No evidence of regulation (shaded orange)

Sponsor
<u>King's College London.</u>
Clinical Study Protocol

REC Ref number:	06/Q0406/119
EudraCT Number:	2006-002330-38
Study Title:	Randomised controlled trial of anti-CD20 in patients with C4d+ Chronic Allograft Nephropathy ('RituxiCAN-C4')
Investigational Product:	Rituximab (MabThera)
Protocol Version:	10 (01-05-2013)

Chief investigator:	Anthony Dorling
Investigator's address:	Dept. of Nephrology and Transplantation, King's College London, Guy's Hospital, 5th Floor Bermondsey Wing, Guy's Hospital, Great Maze Pond, London SE1 9RT
Study Location:	Guy's Hospital, London SE1 9RT
Telephone:	A Dorling 020 7188 8711
Study Sponsor:	King's College London.
Sponsor contact	Jackie Pullen
	Joint Clinical Trials Office, Guys Hospital, Great Maze Pond, London SE19RT. Tel: 020 7188 5732
Medical Contact on site:	Anthony Dorling

1 STUDY SYNOPSIS

Title of clinical trial	A randomised controlled trial of anti-CD20 in C4d+ chronic allograft nephropathy
Sponsor name	King's College London
Eudra CT number for proposed trial	2006-002330-38
Medical condition or disease under investigation	C4d+/- chronic allograft nephropathy (CAN)
Purpose of clinical trial	Evaluate the effectiveness of rituximab in C4d+/- CAN
Primary objective	To determine whether anti-CD20 therapy can stabilise or improve renal function and/or proteinuria in patients with C4d+/-, chronic (humoral) rejection in whom standard therapeutic approaches have failed.
Secondary objective (s)	To compare patient and graft survival between control and rituximab-treated groups<br>To evaluate the adverse effect profile of rituximab in this group<br>To correlate changes in circulating B cell numbers, anti-HLA and non-HLA Ab profiles and titre with responses to standard therapy and / or rituximab<br>To correlate changes in T cell responsiveness to alloantigens with responses to standard therapy and / or rituximab
Study Design	Prospective, randomised, two arm, open-labelled
Study Endpoints	1° - Rate of deterioration in renal function, defined by slope of reciprocal creatinine plot, on samples taken 3-5 months post-randomisation.<br>-Change in degree of proteinuria, where present, at 3-5 months post-randomisation<br>2° endpoints, determined at 3-5 months post-randomisation and at 1, 2 and 3 years post-recruitment are;<br>•Rate of deterioration in renal function, defined by slope of reciprocal creatinine plot, determined by analysis of samples taken since previous assessment.<br>•Patient survival<br>•Graft survival<br>•Incidence of culture positive infection<br>•Incidence of malignancy<br>•Degree of proteinuria<br>•Changes in circulating CD20+ cells in peripheral blood

	•Changes in anti-graft Ab titres, (measured every 3 months)<br>• Changes in T cell responsiveness to alloantigens (measured every 3 months).
Sample Size	A two group chi-squared test with a 0.05 one-sided significance level will have 80% power to detect the difference between 70% stabilisation on rituximab and 20% stabilisation in the control group if 15 patients reach the primary endpoint (i.e. 30 patients complete phase 2). It is anticipated that approximately 25% of patients recruited to the run-in period will enter the randomisation process that signals the beginning of phase 2. Therefore, approximately 120 patients will be enrolled into the study. In addition, in those participants that received a living donor kidney, these donors will be approached to provide up to 5 samples of blood to help with the in vitro analyses.
Summary of eligibility criteria	Male or female renal allograft recipients 18-70 years of age<br>>6/12 post-transplantation<br>Either deteriorating allograft function on reciprocal creatinine plot or significant proteinuria or both.<br>C4d+/- CAN on renal allograft biopsy
Investigational medicinal product and dosage	Rituximab, 1g on day 0 and 1g on day 14
Active comparator product(s)	None
Route(s) of administration	Intravenous infusion
Maximum duration of treatment of a subject	14 days with rituximab. The treatment arms of the study, including optimisation period, formal run-in and post-randomisation phase lasts for 10 months post-recruitment.
Procedures: Screening & enrolment	Potentially eligible patients will be identified by screening renal allograft biopsies performed for 'creeping creatinine' and/or proteinuria. Eligible patients will be asked for informed consent prior to enrolment.
Baseline	In addition to routine tests, blood will be obtained for anti-HLA and non-HLA antibody analysis and for peripheral blood mononuclear cell (PBMC) purification.
Treatment period	The treatment period comprises a 3-month run-in period during which the patient will be on stabilised optimal immunosuppressive therapy. This period will be preceded by up to 2 months to allow tailored-optimisation for that individual. During the formal run-in, patients will be reviewed at least six times in their normal transplant clinic appointments for routine blood biochemistry, full blood

	count and urine analysis. At the end of the run-in period, further blood will be taken for anti-graft antibody analysis and PBMC purification. Those patients in whom allograft function stabilises and/or proteinuria improves will have normal transplant clinic follow-up appointments and have blood taken for further anti-graft antibody and PBMC purification up to every 3 months for 3 years. Those with continued deterioration in either allograft function or persisting or worsening proteinuria will be eligible for randomisation if they consent to phase 2 of the study.<br>These patients will be reviewed during their normal transplant clinic appointments until the primary end-point and will need to have at least 6 routine blood biochemistry, full blood count and urine analysis during the final 3 months of this period, post-randomisation. At the primary end-point, further blood will be taken for anti-graft antibody analysis and PBMC.
End of Study	Follow up will continue for 3 years, with blood taken for anti-graft antibody analysis and PBMC purification every three months
Procedures for safety monitoring during trial	Regular patient interviews and examination, routine haematological and biochemical analyses.<br>Serious adverse events will be reported and forwarded to the sponsor, MHRA, REC and Roche as appropriate<br>The King's transplant research committee will discuss the trial and any safety concerns at their regular three monthly meetings.<br>The external data monitoring committee will review data at 3 points, following randomisation of 10, 20 and 30 patients.
Criteria for withdrawal of patients on safety grounds	Serious adverse effects related to rituximab infusion
Regulatory submissions on safety grounds	

2 STUDY FLOW CHART

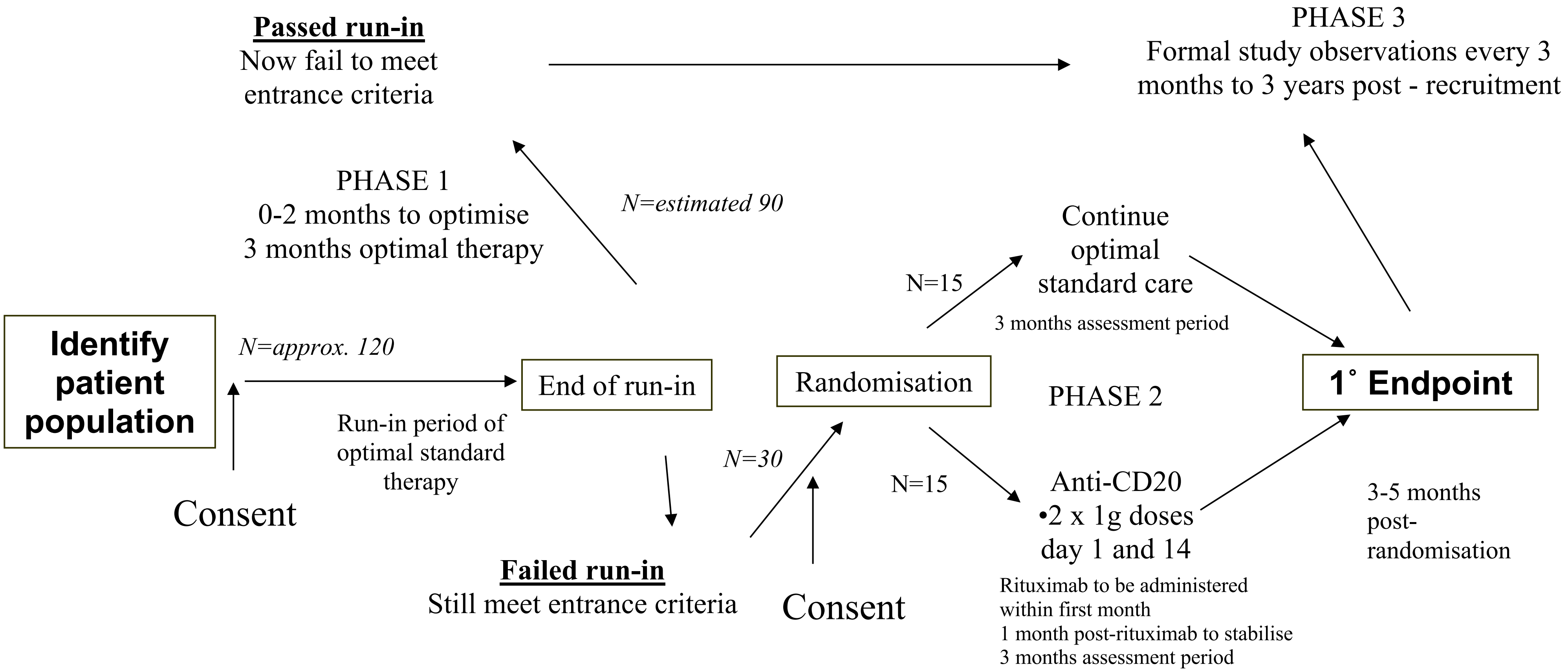

3 TABLE OF CONTENTS

1 STUDY SYNOPSIS 24

2 STUDY FLOW CHART 27

3 TABLE OF CONTENTS 28

4 INTRODUCTION 32

4.1 Background 32

4.2 Clinical Data 33
4.2.1 Efficacy 33
4.2.2 Safety and tolerability 34
4.2.3 Pharmacokinetics & pharmacodynamics 34
4.2.4 Mechanism of action 35

5 RATIONALE FOR STUDY 35

6 TRIAL OBJECTIVE AND PURPOSE 35

7 TRIAL DESIGN 36

7.1 Statement of design 36

7.2 Number of Centres 36

7.3 Number of Subjects 36

7.4 Sample size determination 36

7.5 Randomisation (and blinding) 36

7.6 Study duration 37

7.7 Study objectives 37
7.7.1 Primary objective 37
7.7.2 Secondary objectives 37

7.8 Study endpoints 37
7.8.1 Primary endpoints 37
7.8.2 Secondary endpoints 37

7.9 Trial treatments 38
7.9.1 Optimal 'standard therapy' 38
7.9.2 Patients who fail to stabilise on optimal 'standard therapy' 38

7.10 Criteria for Discontinuation 38
7.10.1 Individual subject 38
7.10.2 Trial 38

8 SELECTION AND WITHDRAWAL OF SUBJECTS 39

8.1 Inclusion Criteria 39

8.2 Exclusion Criteria 39

8.3 Assignment and Randomisation. 39

8.4 Method of Blinding....40

8.5 Subject withdrawal criteria....40
8.5.1 Criteria for withdrawal 40
8.5.2 Follow up of withdrawn subjects 40

9 TREATMENT REGIMENS 40

9.1 Dosage schedules 40
9.1.1 Run-in period....40
9.1.2 'Passed' run-in period 41
9.1.3 'Failed' run-in period 41
9.1.4 Route of Administration....41
9.1.5 Maximum dosage allowed....41
9.1.6 Maximum duration of treatment of a subject 41
9.1.7 Procedures for monitoring subject compliance 42

9.2 Dosage modifications....42

9.3 Legal status of the drug 42

9.4 Drug storage and supply....42

9.5 Concomitant therapy 42

10 STUDY PROCEDURE AND ASSESSMENTS 43

10.1 Screening evaluation 43

10.2 Informed consent....43

10.3 Baseline data 43

10.4 Study assessments....43
10.4.1 Timing of assessments....43
10.4.2 Assessment data, ...44
10.4.3 Renal Biopsy 44

10.5 Post study examination 44

11 EVALUATION OF RESULTS 44

11.1 Response criteria 44
11.1.1 Rate of deterioration of renal function 44
11.1.2 Survival (patient and graft)....45
11.1.3 Infection....45
11.1.4 Malignancy....45
11.1.5 Proteinuria 45
11.1.6 Circulating B cells....45
11.1.7 Anti-graft antibodies....45
11.1.8 Changes in T cell responsiveness....46

12 ASSESSMENT OF SAFETY....46

12.1 Definitions 46

12.1.1 Adverse event 46
12.1.2 Adverse reaction of an investigational medicinal product (AR) 46
12.1.3 Unexpected adverse reaction 46
12.1.4 Serious adverse event or serious adverse reaction 46

12.2 Expected adverse drug reactions: Rituximab 47
12.2.1 Cytokine release syndrome 47
12.2.2 Anaphylaxis 47
12.2.3 Hypotension 47
12.2.4 Cardiac toxicity 47

12.3 Expected Serious Adverse Events-Rituximab 47
12.3.1 General 47
12.3.2 Cardiovascular system 47
12.3.3 Digestive system 47
12.3.4 Blood and lymphatic system 47
12.3.5 Metabolic and nutritional disorders 47
12.3.6 Musculo-skeletal system 47
12.3.7 Nervous system 48
12.3.8 Respiratory system 48
12.3.9 Skin and appendages 48
12.3.10 Others 48
12.3.11 Adverse events less common than 1% 48

12.4 Recording and evaluation of adverse events 48
12.4.1 Assessment of seriousness 48
12.4.2 Assessment of causality 48

12.5 Reporting adverse events 49
12.5.1 Who should report and whom to report to? 49
12.5.2 When to report? 49
12.5.3 How to report? 49

12.6 Responsibilities of Principal Investigators (PIs) and Chief Investigator (CI) in multicentre trial (see figure 1) 50
12.6.1 Reporting times 50
12.6.2 Figure 1 51

13 STATISTICS 52

13.1 Study statistician 52

13.2 Statistical methods to be employed 52

13.3 Interim analyses 52

13.4 Number of Subjects to be enrolled 52

13.5 Criteria for the termination of recruitment 52

13.6 Definition of the end of the trial 52

14 DIRECT ACCESS TO SOURCE DATA / DOCUMENTS 52

15 ETHICAL CONSIDERATIONS 52

15.1 Consent 53

15.2 Ethical committee review 53

15.3 External Data Monitoring Committee 53

15.4 Quality Assurance 53

15.5 Declaration of Helsinki Good Clinical Practise 53

16 DATA HANDLING AND RECORD KEEPING 53

17 FINANCIAL AND INSURANCE 53

18 PUBLICATIONS POLICY 54

19 SUPPLEMENTS 54

19.1 References 54

4 Introduction

4.1 Background

The rate of acute renal allograft loss over the last decade has improved significantly with newer immunosuppressive drugs, but loss due to chronic allograft nephropathy (CAN) remains an important problem; - by 10-12 years after transplantation, approximately 50% of patients have returned to dialysis. Premature allograft failure is now one of the leading causes of end stage renal disease, so that, for instance, almost 5000 patients return to dialysis each year in the USA (3). Although multiple factors contribute to the aetiology of CAN (4), a significant proportion of cases have evidence of ongoing immunologically-mediated damage, or 'chronic rejection' (CR). This may be manifest in glomeruli (transplant glomerulopathy), peritubular capillaries (PTC) or arteries (transplant arteriopathy). The arteriopathy of CR is characterised by neointimal expansion and remodelling, leading to progressive obliteration of the vessel lumen and chronic ischaemia. The pathophysiology of CR is complex and incompletely understood, but a potentially important factor in a sizeable proportion of patients is that they develop anti-HLA antibodies (Ab) prior to suffering graft deterioration (5, 6). If these Ab are donor (graft) specific Ab (DSA), they may bind donor endothelial cells (EC) and if complement-fixing, mediate organ damage in a similar way to acute humoral rejection (7, 8).

The criteria for formal definition of chronic humoral rejection (CHR) includes the detection of circulating DSA in the peripheral blood (9), although by this definition, a significant proportion of patients with antibody-mediated pathology probably go undetected. This is because it has been hypothesised that most high affinity DSA will be deposited within the graft vasculature and DSA within the circulation will only be detected once the antigen binding sites in the graft have been saturated, which will occur only in those generating significant quantities of Ab (5). Although DSA bound within the graft are difficult to detect directly by conventional immunohistology, indirect evidence of their deposition, if they are complement-fixing, is provided by finding complement component C4d deposition on graft PTC EC (9). C4d deposition correlates strongly with ongoing anti-HLA Ab-mediated CHR (10, 11). Therefore, for the purposes of this study, CHR will be defined primarily on the basis of positive PTC C4d staining. By this definition, CHR probably accounts for 1/3rd of all grafts lost to CAN (11).

There is no established treatment for CHR. Theruvath et al reported short-term stabilisation of renal function in 3 out of 4 patients after establishing them on tacrolimus, mycophenolate mofetil (MMF) and prednisolone, a regime based on a one shown to have efficacy in acute humoral rejection (12). Although there are several problems with this approach, the most important is that it only appears to be effective in about 75% of patients, and 25% to continue to reject their grafts. This has been our experience at the Hammersmith, using a similar approach (without steroids), where we have achieved short-term stabilisation of function in a similar proportion of a small cohort of patients.

At the moment, in these patients who continue to deteriorate, we administer another drug called rituximab (brand name Mabthera), because there are good scientific and clinical reasons to believe that it may be beneficial in patients who have evidence of this type of antibody-mediated damage. Rituximab is a chimeric mouse/human monoclonal antibody specific for CD20, a marker found only on certain types of B cells. It is licensed for use in B cell lymphomas, because it is known to deplete B cells and has a relatively benign side effect profile, characteristics which have helped it to quickly become an established therapy for these conditions.

Our experience to date, having administered it to 4 patients, suggests that graft function stabilises in approximately 50% of patients given rituximab. However, we have no firm evidence that these 50% are stabilising because of the rituximab, or whether they would have stabilised anyway (because they needed more time on 'optimal immunosuppression'), nor whether the use of rituximab is going to lead to the development of any longer term problems. Hence, we have designed this study to answer three questions. First, is it the rituximab that stabilises kidney function or will stabilisation occur after staying on optimal treatment for a longer period of time. Second, is the use of rituximab associated with any short, medium and longer-term adverse effects

compared to staying on optimal immunosuppression. Finally, can we monitor the way the immune system responds after each of these treatment approaches, not only to try and understand how the treatments may be working, but also to develop tests that might predict responsiveness to the different types of therapy?

4.2 Clinical Data

4.2.1 *Efficacy*

Rituximab was first approved by the FDA in 1997 for treatment of relapsed or refractory indolent CD20-positive non-Hodgkin's lymphoma (NHL) and has now been shown to be efficacious and well-tolerated in the treatment of NHL and chronic lymphocytic leukaemia with evidence of efficacy in other B cell diseases such as multiple myeloma, Waldenstrom's macroglobulinaemia and post-transplant lymphoproliferative disease (PTLD). Over half a million patients have received rituximab, which means that there is now a well-established safety profile for its use in B cell malignancies for first line and maintenance therapy, when used as monotherapy and in combination with both chemotherapy and immunotherapy.

More recently, rituximab has been used as treatment in autoimmune diseases such as rheumatoid arthritis (RA). The first open-label study used rituximab in combination with cyclophosphamide and steroids in refractory RA in five patients (13). It reported dramatically improved clinical status that was maintained at one year. Since then a double-blind randomised controlled trial involving 161 patients has demonstrated the efficacy of combination therapy with rituximab and methotrexate (MTX) or cyclophosphamide over MTX alone (14). In this study, where the primary endpoint was achievement of an ACR (American College of Rheumatology) response of 50 at 24 weeks, rituximab added to MTX delivered a response in 43% whereas rituximab added to cyclophosphamide delivered a response in 41%, both of which were statistically significantly different to the 13% who responded to MTX alone. There was profound B cell depletion in patients treated with rituximab, a rapid decline in rheumatoid factor levels, but no significant change in immunoglobulin levels and no significant difference in infection rates between the treatment arms.

Rituximab has also been used in systemic lupus erythematosus (SLE), in which B cells are know to play an important role in disease pathogenesis (15, 16). As yet, there have been no randomised controlled trials or head-to-head comparison with conventional immunosuppression in SLE. An early phase I/II study used rituximab as monotherapy at low, medium and full-dose (2 doses of 1000mg 2 weeks apart) in 17 patients (17). This preliminary study demonstrated that in the 11 patients who had good B cell depletion, there was a significant improvement in symptoms and in their Systemic Lupus Activity Measurement Score. Those who did not achieve good B cell depletion did not improve clinically and were also at higher risk of developing human anti-chimeric antibody. 3 patients had serious infections, although these were not felt to be directly related to the treatment with rituximab, but rather to the pre-existing leukopenia of these patients. Another early open label study conducted at University College London in 6 patients who had not responded to conventional treatment for SLE showed that use of medium dose rituximab (2 doses of 500mg) in combination with cyclophosphamide and prednisolone was well-tolerated with no significant adverse events and associated with a sustained clinical improvement (18).

In the context of renal transplantation, rituximab has been reported to successfully treat acute humoral rejection refractory to conventional treatment and to reduce the titres of anti-blood group or anti-HLA antibodies in patients prior to transplantation (19). These data have all come from relatively small groups of patients.

4.2.2 Safety and tolerability

The tolerability and safety of rituximab in haematological malignancy has been well-described in the pivotal phase III study in 166 patients with indolent lymphoma (20) and in patients with other B cell malignancies (21-23).

The most common adverse events were infusion-related reactions, with the majority of patients experiencing mild to moderate symptoms on the first dose, most frequently fever (43%), chills (28%), nausea (18%) and headache (14%) within 2 hours of the infusion. The frequency and severity are reduced by premedication with paracetamol and an antihistamine given 30 to 60 minutes prior to the infusion. These minor reactions remit rapidly on temporary discontinuation of the infusion, and if additional pre-medication is given and the infusion resumed at a lower rate there is a low risk of further reactions. In approximately 10% of patients receiving their first dose hypotension, bronchospasm or angio-oedema may occur, necessitating other supportive and symptomatic treatment. The frequency and severity of adverse events is markedly reduced on subsequent infusions, possibly because of a lower tumour burden.

Severe adverse events are uncommon, with 12% of patients experiencing grade 3 severity and 3% with grade 4 severity. The more severe events which have been reported include pulmonary infiltrates, an interstitial pneumonia, acute respiratory distress syndrome and myocardial infarction / cardiac arrhythmias all occurring within hours of starting the infusion. These events appear to be the result of cytokine release syndrome, related to the rapid lysis of tumour cells. Fatalities have been rarely reported, with the risk of death from an infusion-related anaphylactic-type response (hypotension, angioedema, bronchospasm and hypoxia) of 0.04-0.07%. Those with underlying pulmonary or cardiac disorders are at increased risk of adverse events, and require careful attention to fluid balance, and a reduced infusion rate and additional monitoring may be indicated.

In autoimmune disease, infusion-related reactions were less common than seen in patients with haematological malignancy. So, for instance, in the double-blind placebo-controlled study of 161 patients with RA mentioned above, 36% of patients given rituximab developed a reaction after the first dose compared to 30% of patients given placebo (24). Side effects included transient hypotension/hypertension, pruritus, flushing, rash, fever and dyspnoea. With second and subsequent infusions there was no significant difference between rituximab and placebo (17% and 15%). These rates are significantly lower than the frequency of adverse events in the pivotal study on patients with indolent lymphoma in which 74% experienced an adverse event (20), which may be due to the lack of tumour burden and / or treatment with steroids being part of the trial protocol in the rheumatoid arthritis patients.

4.2.3 Pharmacokinetics & pharmacodynamics

The following is taken from the Core data sheet for MabThera

'Pharmacokinetic studies performed in a phase I study in which patients (N=15) with relapsed B–cell lymphoma were given single doses of rituximab at 10, 50, 100 or 500 mg/m2 indicated that serum levels and half-life of rituximab were proportional to dose [26, 27].

In a cohort of 14 patients among the 166 patients with relapsed or chemoresistant low-grade or follicular non-Hodgkin's lymphoma enrolled in the phase III pivotal trial and given rituximab 375 mg/m^2 as an IV infusion for 4 weekly doses, the mean serum half-life was 76.3 hours (range, 31.5 to 152.6 hours) after the first infusion and 205.8 hours (range, 83.9 to 407.0 hours) after the fourth infusion. The mean C_{max} after the first and fourth infusion were 205.6 ±59.9 µg/mL and 464.7 ±119.0 µg/mL, respectively. The mean plasma clearance after the first and fourth infusion was 0.0382 ±0.0182 L/h and 0.0092 ± 0.0033 L/h, respectively. However, variability in serum levels was large. Rituximab serum concentrations were statistically significantly higher in responding patients than in non-responding patients just prior to and after the fourth infusion and post-treatment. Serum concentrations were negatively correlated with tumor burden and the number of circulating B-cells at baseline. Typically, rituximab was detectable for 3 – 6 months after

administration of the last infusion (25).

Distribution and elimination have not been extensively studied in patients with DLCL, but available data indicate that serum levels of rituximab in DLCL patients are comparable to those in patients with low-grade or follicular NHL following treatment with similar doses."
Little data is available from other patient groups. Pescovitz reports on a single dose study in patients with renal failure (26), in which the half-life was significantly prolonged compared to that above, at up to 14 days. In this group, Rituximab was detectable on the circulation for many months post-dose. In rheumatoid arthritis, the half-life of the second dose has been reported to be as long as 20 days (26).

4.2.4 Mechanism of action

CD20 is a transmembrane protein found predominantly on B cells, specifically on pre-B and mature B cells, including malignant B cells, but not on haematopoietic stem cells, plasma cells and normal tissue. It is vital for B cell differentiation and proliferation. After administration, rituximab appears to cause loss of CD20-positive cells by antibody dependent cellular cytotoxicity, complement-mediated lysis and by direct induction of B cell apoptosis. For the treatment of haematological malignancy, the standard treatment course is 4 doses over 4 weeks, following which there is significant depletion of B cells in peripheral blood lasting approximately 2 to 6 months, with gradual recovery of non-malignant B cell numbers to normal levels by 12 months. However, there is no reduction in the number of plasma cells, serum immunoglobulin levels are well-maintained, and T-cells are completely unaffected.
It is perhaps because of this that rituximab appears not to be associated with the potentially severe immunosuppressive side effects associated with non-selective depleting polyclonal Ab such as anti-lymphocyte or anti-thymocyte globulin, or the mouse monoclonal antibody OKT3. A small percentage of patients (1% approx) have been described to develop human anti-chimeric Ab but not human anti-mouse Ab.

5 RATIONALE FOR STUDY

There is no firmly established treatment for CHR, though as indicated above, short-term stabilisation of renal function appears to result in 75% of patients after conversion to (or optimisation of) MMF and tacrolimus therapy ± prednisolone. Of the 25% who continue to deteriorate, our experience suggests that rituximab can stabilise function in approximately half of these.
Although the use of rituximab in this group is rational, we have no firm evidence of efficacy in this group of patients and no clear idea about whether it's use may be accompanied by a increased risk of acute viral, bacterial or fungal infection or malignancy. This study will address these two points. Additionally, we will perform studies to try and understand how rituximab may be working and to correlate responses to optimal immunotherapy or rituximab with assays of immune responsiveness and histological appearances.
Our ultimate aim is to establish the basis for using rituximab as a first-line therapy in this condition. If this study confirms that it is successful at stabilising function in those patients who fail standard therapy, with an acceptable safety profile, this would pave the way for earlier use of rituximab in this group of patients.

6 TRIAL OBJECTIVE AND PURPOSE

Primary Aim; To establish the efficacy of rituximab at stabilising renal function and/or reducing proteinuria in patients with CHR who have failed to stabilise on standard, optimal therapy

Secondary Aims;
To compare patient and graft survival between control and rituximab-treated groups
To document the adverse effect profile of rituximab in this group
To correlate laboratory parameters with responses to standard therapy or rituximab

7 TRIAL DESIGN

7.1 Statement of design

This is to be an open, controlled randomised study. All eligible patients will be recruited over a three year period, and initially undergo a run-in period during which time standard therapy will be optimised (0-2 months) followed by 3 months on fully optimised therapy. At the end of the run-in, graft function and degree of proteinuria will be re-assessed and patients who still meet the criteria for entrance into the study (i.e. those that still have deteriorating function or persisting or worsening proteinuria) will be randomised to either remain on standard therapy only, or to receive rituximab. For those receiving rituximab, the two infusions will be given within one month of randomisation and the formal 3-month analysis period will begin one month after the last dose of rituximab. For those randomised to stay on standard therapy, the formal 3-month analysis period will begin on the day of randomisation. For both groups, the primary end-point is at the end of the 3-month analysis period, at which point graft function and proteinuria will be analysed. At this point, the period of defined intervention will cease and patient treatment beyond this will be decided solely on clinical grounds, though observations will continue on individual patients for up to three years post-recruitment.

7.2 Number of Centres

Multi-centre trial coordinated from King's College London, Guy's Hospital campus.

7.3 Number of Subjects

Study requires 30 patients to complete phase 2 and this will mean recruiting approximately 120 into the run-in period (phase 1).

7.4 Sample size determination

It is expected that up to 20% of the group randomised to remain on standard treatment may stabilise (because stabilisation on standard therapy may occur beyond the run-in period), whereas it is anticipated that approximately 70% of those receiving rituximab will stabilise. Based on these estimations, at least 15 patients, randomised to each arm will need to reach the primary endpoint to detect these differences at 80% power and 5% significance. In other words, 30 patients recruited to the randomisation phase of the study will need to reach the primary endpoint. Assuming that 25% of the patients recruited to the run-in phase will fail to stabilise by the end of the run-in period, it is estimated that approximately 120 patients will need to be enrolled.

7.5 Randomisation (and blinding)

Randomisation will be by permuted block method with concealment of block size. Randomisation will be stratified by graft function and proteinuria to ensure equal numbers with deteriorating function and isolated proteinuria are allocated to each arm.
On local sites, the PI will evaluate patient progress after the run-in period, allocate appropriate patients to the 'continued deterioration' group, obtain patient consent to progress to phase 2, and then contact the Chief Investigator who will allocate the patient to the appropriate arm of phase 2 (see 8.3).

7.6 Study duration

Each subject will be recruited into the run-in phase (phase 1). This will consist of an initial phase lasting 1-2 months, during which standard treatments will be optimised, followed by a period of 3 months at optimal doses. At the end of this period, participants will have either 'passed' run-in (i.e. stabilised graft function and/or reduced proteinuria), in which case they will be followed for a further 31-32 months (i.e. pass straight to phase 3) (total = 3 years post-recruitment), or 'failed' run-in, in which case they will be eligible for phase 2. The primary endpoint is at the end of a formal 3-month analysis period for each group, which begins on the day of randomisation for the control group and one month after the last dose of rituximab in the rituximab group, though all patients will be followed up for a total of 3 years post-recruitment.

7.7 Study objectives

7.7.1 Primary objective

To determine whether anti-CD20 therapy can stabilise or improve renal function and/or reduce proteinuria in patients with C4d+/-, chronic (humoral) rejection in whom standard therapeutic approaches have failed.

7.7.2 Secondary objectives

To compare patient and graft survival between control and rituximab-treated groups
To evaluate the adverse effect profile of rituximab in this group
To correlate changes in circulating B cell numbers, anti-HLA and non-HLA Ab profiles and titre with responses to standard therapy and / or rituximab
To correlate changes in T cell responsiveness to alloantigens with responses to standard therapy and / or rituximab

7.8 Study endpoints

7.8.1 Primary endpoints

Determined 3-5 months post-randomisation
•Rate of deterioration in renal function, defined by slope of reciprocal creatinine plot, on samples taken in the preceding 3 months.
•Change in degree of proteinuria, where present

7.8.2 Secondary endpoints

Secondary endpoints will be determined at 3-5 months post-randomisation and at 1, 2 and 3 years post-recruitment;
• Rate of deterioration in renal function, defined by slope of reciprocal creatinine plot, using samples taken since the previous assessment
• Patient survival
• Graft survival
• Incidence of culture positive infection
• Incidence of malignancy
• Change in degree of proteinuria, where present
• Changes in circulating CD20+ cells in peripheral blood (measured monthly after rituximab until numbers recovered).
• Changes in anti-graft Ab titres (measured every three months).
• Changes in T cell responsiveness to alloantigens (measured every three months).

7.9 Trial treatments

7.9.1 Optimal 'standard therapy'

During the initial phase of the run-in period, the following standard clinical therapies will be introduced and/or optimised according to the following guidelines;

• Mycophenolate mofetil bd, or enteric coated mycophenolic acid bd, with dose determined according to local unit guidelines. In those centres monitoring MPA levels, dose will be titrated to achieve plasma 12-hour post-dose levels of 1.6-2.75. In these centres, the starting dose will be 500mg bd in patients not already on MMF
• Tacrolimus bd titrated to achieve 12-hour post-dose levels of 4-8. Starting dose 0.05mg/kg bd in patients not already on Tacrolimus
• Statin therapy to achieve total non-fasting cholesterol to ≤ 4.5
• ACE-I and ARB combination therapy to achieve a target bp of ≤140/≤80

Therapy will be introduced on an intention to treat basis and tailored precisely to the individual patient, according to compliance, tolerance and achievement of target levels.

7.9.2 Patients who fail to stabilise on optimal 'standard therapy'

Patients with continuing deterioration or persisting or worsening proteinuria or both who get randomised to the control arm will remain on their individualised optimal therapy.
Patients randomised to the rituximab arm will remain on their individualised optimal therapy and receive two intravenous infusions of rituximab (1g each) 14 days apart, so that the last dose is given as early as possible but definitely within 1-month post-randomisation.

All drugs will be supplied in standard packaging and labelled by the appropriate hospital pharmacy in the same way as all other drugs.

7.10 Criteria for Discontinuation

7.10.1 Individual subject

Subjects can withdraw at any time if they wish. Failure to tolerate one or more components of the standard therapy in the run in period will ***not*** be seen as a reason to discontinue the trial but will be anticipated as an integral part of individualising therapy.
Patients will not receive a second dose of rituximab if they suffer one of the following adverse effects following the first dose: pulmonary infiltrates, interstitial pneumonia, acute respiratory distress syndrome, myocardial infarction, cardiac arrhythmia, hypotension, angioedema, bronchospasm, hypoxia. These patients will continue to be monitored as if they had received both doses in the rituximab arm.

7.10.2 Trial

The randomisation to receive rituximab will be halted temporarily if any one of the following occurs;
• a patient death attributable to rituximab infusion.
• unacceptable incidence of severe side effects attributable to rituximab infusion (see 7.10.1 – if occurring in >10% patients).
In these instances, the trial will undergo urgent review by the External data monitoring committee.

The randomisation to stay on standard therapy alone will be discontinued if it becomes apparent, on intermittent analysis of the primary endpoint data, that there is a highly statistically significant difference in response rates in those patients receiving rituximab compared to standard therapy alone.

8 SELECTION AND WITHDRAWAL OF SUBJECTS

8.1 Inclusion Criteria

To be included in the study the patient must have:
•A functioning kidney allograft (with estimated (e) GFR by MDRD >20) and be >6/12 post-transplantation
•Deteriorating allograft function as defined by linear regression of reciprocal creatinine plot. Deterioration will be defined as a negative slope over at least the preceding 3 months (with at least 6 creatinines included) with an adjusted r^2 >0.35 and a p value of ≤0.05 compared to horizontal baseline. Deterioration will be confirmed by reduction in Cockroft-Gault (CG) eGFR over the same period (to exclude increases in body mass as a cause of a negative slope on reciprocal creatinine plots) OR significant proteinuria as assessed by a urine protein/creatinine ratio of ≥50.
•CAN, by Banff '97 criteria, or transplant glomerulopathy on renal allograft biopsy performed within 6/12 of enrolment
•Diffuse, linear C4d deposition (on at least 25% of peritubular capillary (PTC) and/or glomerular EC of renal transplant biopsy when assessed by immunoperoxidase OR >50% of PTC (alone) when assessed by immunofluorescence) OR PTCitis OR glomerulitis with combined PTC/g score of ≥2.

8.2 Exclusion Criteria

The presence of any of the following will preclude patient inclusion

•<18 years of age
•suspicion of pregnancy confirmed by positive HCG pregnancy test
•untreated ureteric obstruction on ultrasound of allograft
•history of acute allograft rejection in preceding 3/12
•history of MI in preceding 3/12
•history of malignancy in previous 5 years (excluding tumours limited to skin)
•symptomatic IHD
•recipient of simultaneous pancreas/kidney transplant
•recipient of ABO-incompatible kidney
•recipient who underwent an HLA desensitisation procedure prior to transplantation
•evidence, on examination of renal allograft biopsy specimen, of recurrent or de-novo disease (except IgA deposition in absence of mesangial proliferation)
• evidence, on examination of renal allograft biopsy specimen, of CNI toxicity *IF ACCOMPANIED* by mostly supra-therapeutic CNI trough levels in the 6-month period preceding biopsy.
• documented allergy to mouse or chimeric human/mouse proteins
• HepBsAg+, HCV Ab+, HIV+ or HepBcAb+
• administration of lymphocyte depleting antibody within 3 months of enrolment

8.3 Assignment and Randomisation.

• Patient numbers will be allocated according to the order of recruitment at each site and will include a site-specific prefix.

• For randomisation, sequentially numbered opaque envelopes, sealed by an independent third party, will be held by the chief investigator. The envelopes will be opened after patients have consented to phase 2. Patients will be allocated a randomisation number that will be cross-referenced to the patient number. Randomisation will be stratified by graft function and proteinuria to ensure equal numbers with deteriorating function and isolated proteinuria are allocated to each arm.
• Patients who voluntarily withdraw from phase 2 before the primary endpoint (see 8.5) will be replaced by another patient eligible for phase 2.

8.4 Method of Blinding

Patient and staff will be aware of the treatment.

8.5 Subject withdrawal criteria

8.5.1 Criteria for withdrawal

• Patients are free to withdraw consent at any time during the study.
• Patients will be withdrawn from the study if, during phase 1 or 2, they develop a medical problem that necessitates significant change (reduction or enhancement) in their immunosuppressive medication or prevents them receiving the IMP.
• The cases of patients who die during phase 1 or 2 will be assessed by the external data monitoring committee as soon as possible to determine whether the trial should be halted. If the cause of death of a patient during phase 2 is deemed unrelated to the trial, they may be replaced in the analysis by another eligible patient.
• Patients in phase 3 who return to dialysis or undergo pre-emptive renal transplantation will be withdrawn from the trial.

8.5.2 Follow up of withdrawn subjects

• All patients will undergo routine clinical follow up with appropriate monitoring
• Withdrawal during phase 1 will automatically make patients ineligible for phase 2 and 3, but those withdrawing from phase 2 will continue into phase 3, unless they specifically withdraw from this phase also.
• A patient eligible for phase 2, who refuses to consent to enter this phase, will be followed up through phase 3 unless they specifically withdraw from this phase also.
• Patients who voluntarily withdraw consent for phase 2, at any stage before the primary end-point, will be monitored in phase 3 unless they specifically withdraw from phase 3.
• Patients who voluntarily withdraw from phase 2 before the primary endpoint will be replaced by another patient eligible for phase 2.

9 TREATMENT REGIMENS

All women of childbearing age will be advised at enrolment to use effective contraception during the course of the study to avoid pregnancy.

9.1 Dosage schedules

9.1.1 Run-in period.

All patients will undergo a period of optimisation of standard approved therapy, consisting of treatment with the following drugs, unless specifically contraindicated or prevented by a specific adverse event;
•initiation or maintenance of ACE-1 and / or ARB therapy, along with other anti-hypertensive drugs if required, at appropriate doses, aiming for a target bp of <140/<80.

•initiation or maintenance of statin therapy, aiming for a target random whole-blood cholesterol of ≤ 4.5.
• initiation or maintenance of bd MMF or enteric coated MPA, (titrating 12 hour trough MPA levels to within the range 1.6-2.75 in those centres monitoring MPA levels)
•initiation or maintenance of bd tacrolimus, titrating 12-hour trough levels to within the range 4-8
In addition, those patients who have had a step-up' in their immunosuppression will be given anti-infective prophylaxis according to local unit guidelines.
There are no restrictions on the use of other medications.

9.1.2 *'Passed' run-in period*

All patients maintained as above

9.1.3 *'Failed' run-in period*

Control group – all patients maintained as above
Rituximab group – maintenance of all run-in drugs.
As prophylaxis against a first dose reaction, paracetamol 1g and chlorphenamine (chlorpheniramine) 10mg i.v. will be given to all patients 30 minutes before the first infusion of rituximab. In addition, in those patients with a history of previous exposure to chimeric proteins (such as dacluzimab, basiliximab), hydrocortisone 100mg i.v. will also be given at the same time. Patients will be monitored with pulse, blood pressure, and oxygen saturation during the first dose infusion.
All patients will receive 2 infusions of rituximab, separated by 14 days, at a fixed dose of 1g per dose, diluted in 250-500ml saline. The initial rate is 50mg/hour, escalating after 30 minutes to 100mg/hour, and then increasing by 50 mg/hour to a maximum of 400mg/hour. If side effects appear the rate of infusion should be halved or stopped (after discussion).
NOTE: If bronchospasm, hypoxia, or severe dyspnoea occur the infusion should be stopped immediately and help sought from a senior doctor.
All patients receiving rituximab will receive co-trimoxazole (or an appropriate alternative) prophylaxis for 6 months from the first dose at a dose according to unit protocol.

9.1.4 *Route of Administration*

All run-in drugs given orally
Rituximab given by IV infusion

9.1.5 *Maximum dosage allowed*

Run-in drugs – dosages determined by unit protocols, clinical response and/or need to achieve target levels as stated
For the rituximab - two 1g doses separated by 14 days

9.1.6 *Maximum duration of treatment of a subject*

Run-in drugs will be maintained at the individual-tailored optimal until the primary endpoint, though optimal treatments for each individual may change according to clinical or laboratory indication. Beyond the primary endpoint, dosing regimens and targets will be the responsibility of the individual clinician primarily responsible for patient care.
Maximum of two doses of rituximab over two weeks will be given to the experimental group

9.1.7 *Procedures for monitoring subject compliance*

For run-in drugs, BP, cholesterol and drug trough level monitoring will be performed at each clinic visit.
For rituximab, each dose will be given under direct supervision.

9.2 Dosage modifications

See 9.1 – standard therapy drug doses will be altered according to the criteria already outlined or according to local unit protocol where not determined by study protocol.
In those patients who suffer a severe adverse reaction to first dose of rituximab, the second dose will be omitted. These patients will continue to be followed up as stated.

9.3 Legal status of the drug

Rituximab is a licensed drug, but not for chronic allograft nephropathy and this study will be conducted under a clinical trials authorisation from the MHRA. All the 'standard care' drugs are being used within their licensed indications.

9.4 Drug storage and supply

All run-in drugs will be supplied as is our current clinical practice, by the hospital pharmacy initially and subsequently the patient's local pharmacy.

The Investigator is responsible for ensuring that all IMPs received at the site are inventoried and accounted for throughout the study. The IMP will be dispensed to the clinician and any returns of IMP must be documented on the drug accountability form. IMP will be supplied only to subjects participating in the study. Study drugs must be handled in strict accordance with the protocol and the product label and will be stored in a limited access area or in a locked cabinet under appropriate environmental conditions. Prior to administration, the dose and expiry date will be checked and the lot number noted.

Unused study drug and study drugs returned (if applicable), must be available for verification by the sponsor's site monitor during site monitoring. The destruction of unused study drug (both expired or unexpired) or used returned study drug will have to be authorised by the sponsor, and will be documented. The Sponsor may authorize to destroy on site according to local policy.

9.5 Concomitant therapy

Anti-infective drugs, to provide prophylaxis against infection in patients who have had a 'step-up' in their immunosuppression will be used according to the standard protocol of individual units, except in the case of those receiving rituximab, in which case all patients will receive 6 months prophylaxis with co-trimoxazole (or an appropriate alternative), with the dose determined by individual unit protocol.

10 STUDY PROCEDURE AND ASSESSMENTS

10.1 Screening evaluation

Patients who fulfil the basic entry criteria will be identified at the weekly biopsy meeting or on discussion with the histopathologist. These individuals will have been referred for a biopsy on the basis of either proteinuria or deteriorating renal function, after appropriate exclusion of other causes, and will meet the histopatholgical criteria.

10.2 Informed consent

The notes of patients meeting the histopathological criteria will be reviewed by one of the investigators who will then arrange to see the patient, to address the other inclusion and exclusion criteria. Patients will be given the information sheet and will have the opportunity to return for a second consultation within a few days to give informed consent for recruitment into the study. Patients undergoing randomisation will be consented a second time to ensure they understand the nature of the randomisation process and the difference between the two study arms.

10.3 Baseline data

All patients will have a full medical history taken and a clinical examination .The following are to be recorded:

a) Weight and bp
b) Sex and ethnicity
c) Age and date of birth
d) HLA type and that of donor kidney
e) Any significant past medical history, including cause of renal failure, details of previous transplants and cause of graft loss, evidence of sensitisation pre-transplantation (PRA and antibody specificities if known) and previous exposure to chimeric or mouse proteins and of documented allergy to these (patients with a documented allergy are to be excluded from the study)
f) Full blood count (including platelets and differential white cell count)
g) Biochemical series (including urea, creatinine, uric acid, electrolytes, calcium, alkaline phosphatase, AST, CRP, lipid profile). Serial serum creatinines will be imported into an excel spreadsheet to draw a reciprocal creatinine plot.
h) MDRD eGFR on latest creatinine and CG eGFR estimated on the range of creatinines used to draw the reciprocal creatinine plot
i) 12 lead ECG
j) Protein: creatinine ratio on urine sample
k) Separate blood samples will be taken for non-routine analysis of antibody status and for PBMC separation
l) The renal biopsy will be analysed for non-routine light microscopy, immunofluorescence, immunoperoxidase and electron microscopy.
m) Stored blood, serum or histopathology samples, if available, will be analysed as current samples to get retrospective data on histology and immune characteristics.

10.4 Study assessments

10.4.1 <u>Timing of assessments</u>

Up to the primary end-point, patients will be seen ideally every two weeks for routine clinical assessments, appropriate for all patients who have had a change in medication including in immunosuppressive therapy. Patients entering the randomisation phase will have monthly analyses

of circulating B cell numbers until either the primary endpoint (for patients randomised to continued optimal care) or until B cell numbers have recovered to pre-treatment levels (for those randomised to receive rituximab). Formal study assessments will be performed at the end of the run-in period and at the primary end point and ideally will occur at one of these standard transplant clinic follow-up appointments, rather than at extra visits. Beyond the primary endpoint, routine follow up will be at intervals determined by the clinical condition of the patient. Study assessments will occur every 3 months for 3 years post-recruitment.

10.4.2 Assessment data,

The following are to be recorded during formal study assessments:

a) Weight and bp
b) Age
c) Evidence of drug toxicity, confirmed infection or malignancy during previous 3 months (or since last assessment) and action taken.
d) Abnormalities of the full blood count during previous 3 months (or since last formal assessment) and action taken.
e) Abnormalities of biochemical series (including urea, creatinine, uric acid, electrolytes, calcium, alkaline phosphatase, AST, CRP, lipid profile) during previous 3 months (or since last formal assessment) and action taken. Serial serum creatinines will be imported into an excel spreadsheet to draw a reciprocal creatinine plot.
f) MDRD eGFR on latest creatinine and CG eGFR estimated on the range of creatinines used to draw the updated reciprocal creatinine plot
g) 12 lead ECG
h) Protein: creatinine ratio on urine sample
i) In those patients who have returned to long-term dialysis or re-transplanted, date of first dialysis (or of –re-transplantation) and time from enrolment will be recorded.
j) Separate blood samples will be taken for non-routine analysis of antibody status and for PBMC separation

10.4.3 Renal Biopsy

There is no formal requirement for a renal biopsy at any time during the study, although enrolment does not prevent transplant renal biopsies being performed on clinical grounds, (for example to rule out the development of BK nephropathy in patients who have had immunosuppression increased).

10.5 Post study examination

No formal post-study examination required as data will be available from ongoing routine clinical visits

11 EVALUATION OF RESULTS

11.1 Response criteria

11.1.1 Rate of deterioration of renal function

This will be determined by linear regression analysis of the 1/creatinine plot according to the criteria used by Dudley in the creeping creatinine study (1). This requires analysis of at least 6 data

points over three months, with deterioration defined as a negative slope. To be valid, the linear regression should yield an adjusted r2 value of ≥0.35 and have a p value of ≤0.05 compared to a horizontal baseline. The data obtained at the end of run-in period and subsequently at the primary and secondary endpoints will be compared to that obtained at baseline. A reduced rate of deterioration will be defined as a change in the slope of the curve so that it becomes *less* negative (i.e. it moves towards the horizontal baseline but is still significantly different from the baseline ($p \leq 0.05$). At the end of the run-in period, patients with these data will undergo randomisation. Stabilisation in renal function will be defined as a change in the slope so that it is not significantly different from the horizontal baseline (i.e. $p > 0.05$ on linear regression analysis). An improvement in renal function will be defined as a change in the slope so that it becomes positive. To rule out changes in body mass (which influences plasma creatinine levels) as a cause of the change in slope of the reciprocal creatinine plot, CG eGFR will be performed on the study creatinines at each study assessment.

11.1.2 Survival (patient and graft)

These will be measured from the date of recruitment and will be reported for all deaths and graft failures due to all causes. Graft failure will be defined as the return to long-term dialysis or re-transplantation. The cause of death or graft failure is thus to be recorded in all instances.

11.1.3 Infection

This will be defined as a positive microbiological culture or other test confirming viral, bacterial or fungal replication in association with specific symptoms. Also, clinical episodes with confirmatory imaging of infection (for instance, consolidation on lung imaging) will be regarded as an infective episode and recorded.

11.1.4 Malignancy

This will be defined by histopathological confirmation of malignancy on a biopsy of any suspicious lesion.

11.1.5 Proteinuria

This will be defined by the protein:creatinine ratio of a urine sample and is significant if >50. The data obtained at the end of run-in period and subsequently at the primary and secondary endpoints will be compared to that obtained at baseline. Reduction in proteinuria will be defined by a protein:creatinine ratio that has fallen to <50. Worsening proteinuria will be defined as a protein:creatinine ratio that has increased. Persistent proteinuria will be defined by a protein:creatinine ratio that has remained >50, even if it has changed from the previous measurement. At the end of the run-in period, patients with persisting or worsening proteinuria will undergo randomisation.

11.1.6 Circulating B cells

The number of circulating B cells per unit volume of blood will be determined by flow cytometry using CD20 and/or CD19 as a marker of B cells.

11.1.7 Anti-graft antibodies

Serum will be prepared from 20mls of clotted blood and stored in a dedicated freezer in marked aliquots. The following analyses will be performed; Flow PRA screening and specific antigen tests, Luminex screen, lymphocytotoxic cross match using donor or surrogate donor material, anti-GBM antibodies, anti-MIC antibodies, anti-vimentin antibodies. All these have been associated with CAN and chronic humoral rejection.

11.1.8 Changes in T cell responsiveness

PBMC will be prepared from 50mls of heparinised or EDTA anticoagulated blood using standard laboratory protocols and used in proliferation and cytokine assays with donor or surrogate donor stimulator cells to address the role of B cells and CD25+ regulatory T cells in the allogeneic response.
[NB For subjects that received a living kidney, where the donor is available, separate consent should be sought from the donor to provide blood specifically for this study]

12 ASSESSMENT OF SAFETY

12.1 Definitions

12.1.1 Adverse event

Any untoward medical occurrence in a patient or clinical trial subject administered a medicinal product and which does not necessarily have a causal relationship with this treatment.
An adverse event can therefore be any unfavourable and unintended sign (including an abnormal laboratory finding), symptom, or disease temporally associated with the use of an investigational medicinal product, whether or not considered related to the investigational medicinal product.

12.1.2 Adverse reaction of an investigational medicinal product (AR)

All untoward and unintended responses to an investigational medicinal product related to any dose administered. All adverse events judged by either the reporting investigator or the sponsor as having a reasonable causal relationship to the investigational medicinal product qualify as adverse reactions. The expression reasonable causal relationship means to convey in general that there is evidence or argument to suggest a causal relationship

12.1.3 Unexpected adverse reaction

An adverse reaction, the nature, or severity of which is not consistent with the applicable product information (e.g. investigator's brochure for an unapproved investigational product or summary of product characteristics (SmPC) for an authorised product).
When the outcome of the adverse reaction is not consistent with the applicable product information this adverse reaction should be considered as unexpected.

The term “severe” is often used to describe the intensity (severity) of a specific event. This is not the same as “serious,” which is based on patient/event outcome or action criteria.

12.1.4 Serious adverse event or serious adverse reaction

Any untoward medical occurrence or effect that:

- results in death,
- is life-threatening
- requires hospitalisation or prolongation of existing inpatients´ hospitalisation,
- results in persistent or significant disability or incapacity,
- is a congenital anomaly or birth defect.

Life-threatening in the definition of a serious adverse event or serious adverse reaction refers to an event in which the subject was at risk of death at the time of event; it does not refer to an event which hypothetically might have caused death if it were more severe.

12.2 Expected adverse drug reactions: Rituximab

12.2.1 Cytokine release syndrome

This is characterised by severe dyspnoea, sometimes accompanied by bronchospasm and hypoxia, in addition to fever, chills, rigors, urticaria, and angioedema. In oncological practice, this has been associated with features of the tumour lysis syndrome, such as hyperuricaemia, hyperkalaemia, hypocalcaemia, acute renal failure, elevated LDH and may be associated with acute respiratory failure.

12.2.2 Anaphylaxis

Anaphylaxis can occur, typically within minutes of starting the infusion (which differentiates it from the cytokine release syndrome). It is most likely in patients with human anti-mouse or human anti-chimeric antibody titres (caused by previous exposure to therapeutic antibodies, and hence rituximab will be used in caution, along with appropriate prophylactic therapy (see previously) in individuals exposed to other therapeutic antibodies).

12.2.3 Hypotension

Common during infusion of rituximab in tumour lysis setting.

12.2.4 Cardiac toxicity

Angina and cardiac arrhythmias such as atrial flutter and fibrillation, heart failure or myocardial infarction have been reported.

12.3 Expected Serious Adverse Events-Rituximab

The following are quoted in the SmPC as adverse events reported by at least 1% of 356 patients in clinical trials in the treatment of haematological malignancy. In descending order of frequency:

12.3.1 General

Fever, chills, asthenia, headache, throat irritation, abdominal pain, back pain, flushing, chest pain, malaise, tumour pain, cold syndrome, neck pain.

12.3.2 Cardiovascular system

Hypotension, hypertension, tachycardia, arrhythmia, postural hypotension.

12.3.3 Digestive system

Nausea, vomiting, diarrhoea, dyspepsia, anorexia, dysphagia, stomatitis, constipation.

12.3.4 Blood and lymphatic system

Leukopenia, neutropenia, thrombocytopenia, anaemia.

12.3.5 Metabolic and nutritional disorders

Angioedema, hyperglycaemia, peripheral oedema, LDH increase, hypocalcaemia, face oedema, weight loss.

12.3.6 Musculo-skeletal system

Myalgia, arthralgia, hypertonia, pain.

12.3.7 Nervous system
Dizziness, paraesthesia, anxiety, insomnia, vasodilatation, hypoaesthesia, agitation.

12.3.8 Respiratory system
Bronchospasm, rhinitis, increased cough, dyspnoea, chest pain, respiratory disease.

12.3.9 Skin and appendages
Pruritus, rash, urticaria, night sweats, sweating.

12.3.10 Others
Lacrimation disorder, conjunctivitis, ear pain, tinnitus

12.3.11 Adverse events less common than 1%
Coagulation disorders, asthma, bronchiolitis obliterans, hypoxia, abdominal enlargement, pain at the infusion site, bradycardia, lymphadenopathy, nervousness, depression, dysgeusia.

12.4 Recording and evaluation of adverse events
Individual adverse events should be evaluated by the investigator and, where indicated, they should be reported according to the processes outlined in sections 12.5 and 12.6. This includes the evaluation of its seriousness and the causality between the investigational medicinal product(s) and/or concomitant therapy and the adverse event.
The sponsor, through the chief investigator, has to keep detailed records of all AEs reported to him by the investigator(s´) and to perform an evaluation with respect to seriousness, causality and expectedness.

12.4.1 Assessment of seriousness
Mild: The subject is aware of the event or symptom, but the event or symptom is easily tolerated

Moderate: The subject experiences sufficient discomfort to interfere with or reduce his or her usual level of activity

Severe: Significant impairment of functioning; the subject is unable to carry out usual activities and / or the subject's life is at risk from the event.

12.4.2 Assessment of causality
Probable: A causal relationship is clinically / biologically highly plausible and there is a plausible time sequence between onset of the AE and administration of the investigational medicinal product and there is a reasonable response on withdrawal.

Possible: A causal relationship is clinically / biologically plausible and there is a plausible time sequence between onset of the AE and administration of the investigational medicinal product.

Unlikely: A causal relation is improbable and another documented cause of the AE is most plausible.

Unrelated: A causal relationship can be definitely excluded and another documented cause of the AE is most plausible.

12.5 Reporting adverse events

12.5.1 Who should report and whom to report to?

King's College London have delegated the delivery of the Sponsor's responsibility for Pharmacovigilance (as defined in Regulation 5 of the Medicines for Human Use (Clinical Trials) Regulations 2004 to the Joint Clinical Trials Office (JCTO).

All SAEs, SARs and SUSARs (excepting those specified in this protocol as not requiring reporting) will be reported immediately by the Chief Investigator to the (JCTO) in accordance with the current Pharmacovigilance Policy. The Chief Investigator will provide an annual report of all SARs (expected and unexpected), and SAEs which will be distributed to the sponsor via the JCTO, MHRA and the REC

12.5.2 When to report?

12.5.2.1 Fatal or life-threatening SUSARs

The JCTO will report SUSARs (Suspected Unexpected Serious Adverse Reactions) and other SARs to the regulatory authorities (MHRA, competent authorities of other EEA (European Economic Area) states in which the trial is taking place.

The Chief Investigator will report to the relevant ethics committees. Reporting timelines are as follows:

– SUSARs which are fatal or life-threatening must be reported not later than 7 days after the sponsor is first aware of the reaction. Any additional relevant information must be reported within a further 8 days.

12.5.2.2 Non fatal and non life-threatening SUSARs

– SUSARs that are not fatal or life threatening must be reported within 15 days of the sponsor first becoming aware of the reaction.

The Chief Investigator will provide an annual report of all SARs (expected and unexpected), and SAEs which will be distributed to the Sponsor (JCTO), MHRA and the REC..

12.5.3 How to report?

12.5.3.1 Minimum criteria for initial expedited reporting of SUSARs

Information on the final description and evaluation of an adverse reaction report may not be available within the required time frames for reporting. For regulatory purposes, initial expedited reports should be submitted within the time limits as soon as the minimum following criteria are met:

a) a suspected investigational medicinal product,
b) an identifiable subject (e.g. study subject code number),
c) an adverse event assessed as serious and unexpected, and for which there is a reasonable suspected causal relationship,
d) an identifiable reporting source,
and, when available and applicable:
e) The EudraCT number:
f) An unique case identification (i.e. sponsor's case identification number).

12.6 Responsibilities of Principal Investigators (PIs) and Chief Investigator (CI) in multicentre trial (see figure 1)

• The PI on each secondary site will take responsibility for reporting *all* adverse events and pregnancy to the CI at the primary site.
• All reports should be on the SAE form provided for the study.
• As outlined above, the PIs are to distinguish between' Adverse event' (AE) and 'serious adverse event' (SAE). The definition of 'serious' is contained in section 12.1.4 of the protocol. See the same section for definition of 'life-threatening' SAE's.
• Report all SAEs to CI, including those related to the IMP (rituximab) ***and those*** not related to IMP.
• In deciding whether the AE is **related** to the IMP, the PI must report an AE as related to the IMP if there is a suspected causal relationship to the IMP (if the event is probably or possibly related). See section 12.4.2 above
• Considering SAEs related to the IMP, PIs must distinguish between 'expected' SAE and 'unexpected' SAE. The definitions of 'expected' AEs are contained in sections 12.2 and 12.3 of the protocol. All other serious AEs are therefore 'unexpected'.

12.6.1 Reporting times

• PI on secondary site must report all Suspected Serious AEs (expected or unexpected) related to IMP to CI on primary site as soon as they become aware
• PI on secondary site must report all Serious AEs unrelated to IMP to CI on primary site as soon as they become aware of the event.
• PI on secondary site must report all other AEs, related or unrelated to IMP, to CI on primary site within 28 days of being aware.
• CI at the primary site will take responsibility for informing the JCTO on behalf of the sponsor, Main Research Ethics Committee and other interested parties within the required time frames of any event that require expedited reporting.

12.6.2 Figure 1

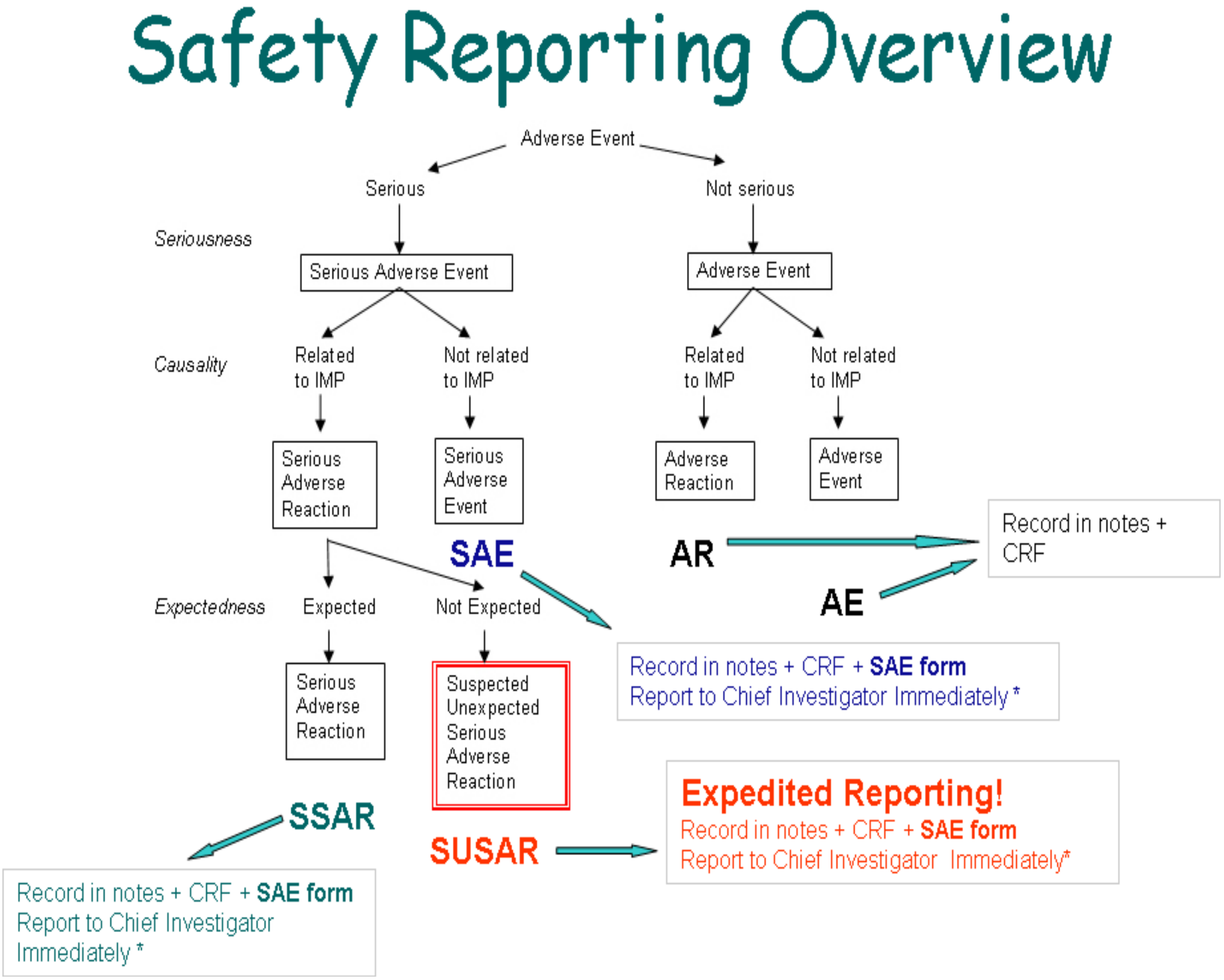

13 STATISTICS

13.1 Study statistician

Irene Rebollo Mesa, MRC Centre for Transplantation, King's College London.

13.2 Statistical methods to be employed

Response rates in the control and study groups will be compared by Chi squared test
Student's t test for independent samples will be used to compare adverse events and other demographic data
Log rank test will be used to compare patient and graft survival rates

13.3 Interim analyses

An interim analysis will be performed after 30 and 60 patients have been enrolled. The King's College London transplantation research committee will review progress and safety data at each meeting (approximately every two months).

13.4 Number of Subjects to be enrolled

Assuming that 20% of those randomised to the control arm settle spontaneously within the study period, and that 70% of those receiving rituximab stabilise, a two group chi-squared test with a 0.05 one-sided significance level will have 80% power to detect this difference when 15 patients are randomised to each arm. So 30 patients will need to be enrolled into the randomisation phase. Based on the evidence available from small published studies and from the experience at the Hammersmith, it is anticipated that 25% of patients who undergo optimisation of standard immunotherapy will continue to deteriorate and thus be eligible for randomisation. Therefore, it is anticipated that approximately 120 patients will need to enter the run-in period of the study.

13.5 Criteria for the termination of recruitment

•When 30 patients randomised in phase 2 have reached the primary endpoint.

Earlier termination *will be considered* by the External data monitoring committee on the following grounds:

A significant excess of adverse events in the study arm.
A highly significant difference in response rates at interim analysis if one group is faring significantly worse than the other

13.6 Definition of the end of the trial

The trial will be defined as finished when the last patient recruited reaches the end of the three-year follow-up period. Within 90 days of completion, the local research ethics committee and the MHRA will be informed.

14 DIRECT ACCESS TO SOURCE DATA / DOCUMENTS

The Investigator(s) will permit trial-related monitoring, audits, REC review, and regulatory inspections (where appropriate) by providing direct access to source data and other documents

15 ETHICAL CONSIDERATIONS

15.1 Consent

All patients will freely give their informed consent to participate in the study. A patient may decide to withdraw from the study at any time without prejudice to their future care.

15.2 Ethical committee review

The study protocol is to be seen and approved by the appropriate ethical review committee(s) of any participating hospital. Copies of the letters of approval are to be filed in the study file.

15.3 External Data Monitoring Committee

- Consists of Chairman and two members from centres not involved in the trial.
- All will be consultant physicians or surgeons with active clinical practise in renal transplantation.
- Committee will review data and outcomes after randomisation of 10, 20 and 30 patients.
- Committee will review all patient deaths during the course of the trial.

15.4 Quality Assurance

Monitoring of this trial to ensure compliance with Good Clinical Practice and scientific integrity will be managed and oversight retained by the Joint Clinical Trials Office Quality Team.

15.5 Declaration of Helsinki Good Clinical Practise

The study is to be carried out in conformation in accordance with the declaration of Helsinki *(1996),* and Good Clinical Practice as defined in the UK Clinical Trial Regulations.

16 DATA HANDLING AND RECORD KEEPING

All data will be held on a password-protected dedicated PC or laptop with additional back-ups on a password-protected secure server and appropriate magnetic or encrypted optical storage media. During the trial paper copies will be held in a locked filing cabinet in the chief investigators office and retained for 10 years following the end of the study.
All trial data will be stored and archived in line with the Medicines for Human Use (Clinical Trials) Amended Regulations 2006 as defined in the Joint Clinical Trials Office Archiving SOP Record keeping will be the responsibility of the investigators.

17 FINANCIAL AND INSURANCE

The study will be indemnified by King's College London for negligent and non-negligent harm. In addition, Professor Dorling also has independent insurance with medical defence societies.

A fellowship from the Medical Research Council UK provides salary for the trial Research Fellow and also provides funds to perform some of the non-routine analyses that are an integral part of this project. Additional funds for other non-routine analyses have also been provided by the Roche Organ Transplantation Research Foundation, specifically to look for non-HLA-specific antibodies and to perform the PBMC and T cell analyses. Roche have agreed to supply the IMP (rituximab) free of charge.

18 PUBLICATIONS POLICY

The chief investigator will review all presentations and publications arising from this study and decide authorship in accordance with accepted guidelines. Roche, as providers of the study drug, will be notified of any such material and have a right to view prior to presentation or submission.

19 SUPPLEMENTS

19.1 References

1. Dudley C, Pohanka E, Riad H, Dedochova J, Wijngaard P, Sutter C, et al. Mycophenolate mofetil substitution for cyclosporine a in renal transplant recipients with chronic progressive allograft dysfunction: the "creeping creatinine" study. *Transplantation* (2005) 79(4):466-75. PubMed PMID: 15729174.
2. Galliford J, Cook HT, Brookes PA, Chan K, Taube D, Dorling A. Reversal of refractory c4d chronic allograft nephropathy with rituximab. *Amer J Transplantation* (2007) 7(Suppl. 2):526-.
3. USRDS. Annual Data Report: Atlas of End-Stage Renal Disease in the United States. National Institutes if Health, National Institute of Diabetes and Digestive and Kidney Diseases, Bethesda, MD, (2004).
4. Pascual M, Theruvath T, Kawai T, Tolkoff-Rubin N, Cosimi AB. Strategies to improve long-term outcomes after renal transplantation. *N Engl J Med* (2002) 346(8):580-90. Epub 2002/02/22. doi: 10.1056/NEJMra011295. PubMed PMID: 11856798.
5. Terasaki PI. Humoral theory of transplantation. *Am J Transplant* (2003) 3(6):665-73. Epub 2003/06/05. PubMed PMID: 12780557.
6. Davenport A, Younie ME, Parsons JE, Klouda PT. Development of cytotoxic antibodies following renal allograft transplantation is associated with reduced graft survival due to chronic vascular rejection. *Nephrol Dial Transplant* (1994) 9(9):1315-9. PubMed PMID: 7816298.
7. Halloran PF, Wadgymar A, Ritchie S, Falk J, Solez K, Srinivasa NS. The significance of the anti-class I antibody response. I. Clinical and pathologic features of anti-class I-mediated rejection. *Transplantation* (1990) 49(1):85-91. PubMed PMID: 2301035.
8. Lee PC, Terasaki PI, Takemoto SK, Lee PH, Hung CJ, Chen YL, et al. All chronic rejection failures of kidney transplants were preceded by the development of HLA antibodies. *Transplantation* (2002) 74(8):1192-4. PubMed PMID: 12438971.
9. Racusen LC, Colvin RB, Solez K, Mihatsch MJ, Halloran PF, Campbell PM, et al. Antibody-mediated rejection criteria - an addition to the Banff 97 classification of renal allograft rejection. *Am J Transplant* (2003) 3(6):708-14. PubMed PMID: 12780562.
10. Mauiyyedi S, Pelle PD, Saidman S, Collins AB, Pascual M, Tolkoff-Rubin NE, et al. Chronic humoral rejection: identification of antibody-mediated chronic renal allograft rejection by C4d deposits in peritubular capillaries. *J Am Soc Nephrol* (2001) 12(3):574-82. PubMed PMID: 11181806.
11. Regele H, Bohmig GA, Habicht A, Gollowitzer D, Schillinger M, Rockenschaub S, et al. Capillary deposition of complement split product C4d in renal allografts is associated with basement membrane injury in peritubular and glomerular capillaries: a contribution of humoral immunity to chronic allograft rejection. *J Am Soc Nephrol* (2002) 13(9):2371-80. PubMed PMID: 12191982.

12. Theruvath TP, Saidman SL, Mauiyyedi S, Delmonico FL, Williams WW, Tolkoff-Rubin N, et al. Control of antidonor antibody production with tacrolimus and mycophenolate mofetil in renal allograft recipients with chronic rejection. *Transplantation* (2001) 72(1):77-83. Epub 2001/07/27. PubMed PMID: 11468538.
13. Edwards JC, Cambridge G. Sustained improvement in rheumatoid arthritis following a protocol designed to deplete B lymphocytes. *Rheumatology (Oxford)* (2001) 40(2):205-11. PubMed PMID: 11257159.
14. Edwards JC, Szczepanski L, Szechinski J, Filipowicz-Sosnowska A, Emery P, Close DR, et al. Efficacy of B-cell-targeted therapy with rituximab in patients with rheumatoid arthritis. *N Engl J Med* (2004) 350(25):2572-81. PubMed PMID: 15201414.
15. Lipsky PE. Systemic lupus erythematosus: an autoimmune disease of B cell hyperactivity. *Nat Immunol* (2001) 2(9):764-6. PubMed PMID: 11526379.
16. Looney RJ, Anolik J, Sanz I. B lymphocytes in systemic lupus erythematosus: lessons from therapy targeting B cells. *Lupus* (2004) 13(5):381-90. PubMed PMID: 15230297.
17. Looney RJ, Anolik JH, Campbell D, Felgar RE, Young F, Arend LJ, et al. B cell depletion as a novel treatment for systemic lupus erythematosus: a phase I/II dose-escalation trial of rituximab. *Arthritis Rheum* (2004) 50(8):2580-9. PubMed PMID: 15334472.
18. Leandro MJ, Edwards JC, Cambridge G, Ehrenstein MR, Isenberg DA. An open study of B lymphocyte depletion in systemic lupus erythematosus. *Arthritis Rheum* (2002) 46(10):2673-7. PubMed PMID: 12384926.
19. Salama AD, Pusey CD. Drug insight: rituximab in renal disease and transplantation. *Nature Clinical Practice Nephrology* (2006) 2(4):221-30. PubMed PMID: ISI:000236390800013.
20. McLaughlin P, Grillo-Lopez AJ, Link BK, Levy R, Czuczman MS, Williams ME, et al. Rituximab chimeric anti-CD20 monoclonal antibody therapy for relapsed indolent lymphoma: half of patients respond to a four-dose treatment program. *J Clin Oncol* (1998) 16(8):2825-33. PubMed PMID: 9704735.
21. Plosker GL, Figgitt DP. Rituximab: a review of its use in non-Hodgkin's lymphoma and chronic lymphocytic leukaemia. *Drugs* (2003) 63(8):803-43. PubMed PMID: 12662126.
22. Bosly A, Keating MJ, Stasi R, Bradstock K. Rituximab in B-cell disorders other than non-Hodgkin's lymphoma. *Anticancer Drugs* (2002) 13 Suppl 2:S25-33. PubMed PMID: 12710588.
23. Kimby E. Tolerability and safety of rituximab (MabThera). *Cancer Treat Rev* (2005) 31(6):456-73. PubMed PMID: 16054760.
24. Szczepanski L, Szechinski J, Filipowicz-Sosnowska A, Emery P, Edwards JCW, Magrini F, et al. Safety data from 48 weeks follow-up of a randomised controlled trial of rituximab in patients with rheumatoid arthritis. *Arthritis and Rheumatism* (2003) 48(9):S121-S. PubMed PMID: ISI:000185432800249.
25. Berinstein NL, Grillo-Lopez AJ, White CA, Bence-Bruckler I, Maloney D, Czuczman M, et al. Association of serum Rituximab (IDEC-C2B8) concentration and anti-tumor response in the treatment of recurrent low-grade or follicular non-Hodgkin's lymphoma. *Ann Oncol* (1998) 9(9):995-1001. PubMed PMID: 9818074.
26. Pescovitz MD. Rituximab, an anti-cd20 monoclonal antibody: history and mechanism of action. *Am J Transplant* (2006) 6(5 Pt 1):859-66. PubMed PMID: 16611321.
